# Chromene- and Quinoline-3-Carbaldehydes: Useful Intermediates in the Synthesis of Heterocyclic Scaffolds

**DOI:** 10.3390/molecules25173791

**Published:** 2020-08-20

**Authors:** Djenisa H. A. Rocha, Vasco F. Batista, Emanuel J. F. Balsa, Diana C. G. A. Pinto, Artur M. S. Silva

**Affiliations:** 1 LAQV-REQUIMTE & Department of Chemistry, Campus de Santiago, University of Aveiro, 3810-193 Aveiro, Portugal; djenisa@ua.pt (D.H.A.R.); vfb@ua.pt (V.F.B.); emanuelbalsa@ua.pt (E.J.F.B.); artur.silva@ua.pt (A.M.S.S.); 2 CICECO-Aveiro Institute of Materials & Department of Chemistry, Campus de Santiago, University of Aveiro, 3810-193 Aveiro, Portugal

**Keywords:** chromene-3-carbaldehydes, 3-styryl-2*H*-chromenes, quinoline-3-carbaldehydes, 3*H*-chromeno[3,4-c]quinolines, 3-styrylquinoline-1(2*H*)-carbaldehydes, Wittig reaction

## Abstract

Chromenes and quinolines are recognized as important scaffolds in medicinal chemistry. Herein, the efficient use of chromene- and quinoline-3-carbaldehydes to synthesize other valuable heterocycles is described. These carbaldehydes are obtained in excellent yields through the Vilsmeyer-Haack reaction of flavanones and azaflavanones. Protocols towards the synthesis of new heterocycles, such as 3*H*-chromeno[3–*c*]quinolines, (*Z*/*E*)-2-aryl-4-chloro-3-styryl-2*H*-chromenes, and (*E*)-2-aryl-4-chloro-3-styrylquinoline-1(2*H*)-carbaldehydes were established. Altogether, we demonstrate the value of chromene- and quinoline-3-carbaldehydes as building blocks.

## 1. Introduction

The synthesis of new heterocyclic compounds is a significant aspect of medicinal chemistry due to the recognized value of this type of compound in the development of new drugs. In this regard, quinoline and chromene cores are interesting frameworks owing to their established medicinal value. Biological activities, such as anticancer [1,2], antimicrobial [3,4,5], anti-Alzheimer’s [6], antioxidant [7], and cardiovascular [8], can be highlighted. These nuclei can be found in natural compounds [9,10,11,12] and synthetic materials [13,14,15].

**Scheme 1:**
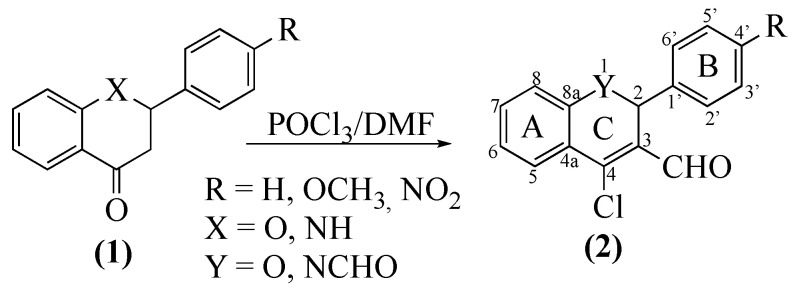
Synthesis of 4-chlorochromene-3-carbaldehydes **2a**–**c** and 4-chloroquinoline-3-carbaldehydes **2d**–**f**.

In this manuscript, the usefulness of 4-chloro(chromene- and quinoline)-3-carbaldehydes in the synthesis of other significant heterocycles is described. Moreover, the 4-chloro(chromene- and quinoline)-3-carbaldehydes **2** are easily obtained, in good to excellent yields, through a Vilsmeyer-Haack reaction (Scheme 1) using flavanones **1** (X = O) and 2-aryl-2,3-dihydroquinolines **1** (X = NH, azaflavanones), respectively. Recently we established a straightforward protocol to achieve, in excellent yields, these flavanone and azaflavanone derivatives [16].

Among the significant heterocycles described are indole and quinoline derivatives recognized bioactive alkaloids, for which-New synthetic methodologies are always essential to be established. Moreover, the study of the use of 4-chloro(chromene- and quinoline)-3-carbaldehydes **2** in Wittig reactions is also described and allowed the synthesis of interesting derivatives bearing a 3-styryl moiety. The work herein reported, besides the diversity of structures obtained, also demonstrates the scope of the transformations.

## 2. Results and Discussion

The Vilsmeyer-Haack formylation is an everyday procedure in our group [17,18] that was easily used in the formylation of flavanones **1** (Scheme 1, X = O) and, in the case of azaflavanones **1**, (Scheme 1, X = NH), required a few optimization steps. The quantities used and the reaction time were adjusted to ensure the total formylation at botH-*N*-1 and C-3 and consequently avoid monoformylated derivatives.

Nuclear magnetic resonance experiments confirmed the structures of both 2-aryl-4-chloro-2*H*-chromene-3-carbaldehydes **2a**–**c** and 2-aryl-4-chloro-1,2-dihydroquinoline-1,3-dicarbaldehydes **2d**–**f**. The most typical features in their ^1^H-NMR spectra are the singlets at δ = 6.33–6.47 and 6.88–7.00 ppm corresponding to the resonance of H-2, respectively, for compounds **2a**–**c** and **2d**–**f**. Other important signals are seen at δ = 10.27–10.32 and 10.30–10.33 ppm due to the formyl protons’ resonances. In the ^13^C-NMR spectra, the signal due to the 3-CHO at δ = 187.6–188.2 ppm and the signal due to C-2 at δ = 73.8–75.0 ppm for compounds **2a**–**c** and at δ = 50.2–50.6 ppm for compounds **2d**–**f** should be highlighted.

The indole nucleus is a prominent constituent of alkaloids such as serotonin and melatonin [19,20] and is also present in many bioactive alkaloids. Antitumoral [21,22], anti-arrhythmic [19], anti-inflammatory [23], antimicrobial [24], and antihypertensive properties are amongst the reported biological activities. The synthesis of indole derivatives is largely explored due to relevant biological applications described for this scaffold, and there are reports on the synthesis of indole hybrids [25,26,27]. We thought it would be interesting to obtain chromene-indole hybrids, so we studied the reaction of 2-aryl-4-chloro-2*H*-chromene-3-carbaldehydes **2a**–**c** with indole (**3**). Unexpectedly, complex mixtures of compounds were obtained. The two major products’ isolation suggested that they were derivatives presenting one and two indole units, respectively.

**Scheme 2:**
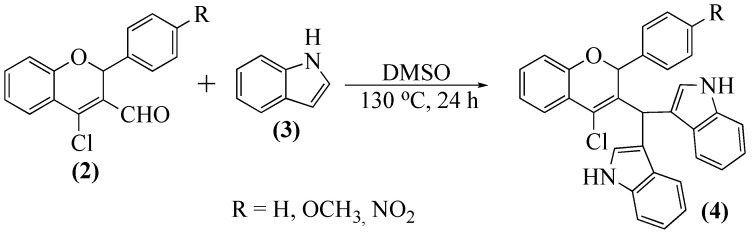
Synthesis of 3,3′-[(2-aryl-4-chloro-2*H*-chromen-3-yl)methylene]bis(1*H*-indoles) **4a**–**c**.

Furthermore, the 2-aryl-4-chloro-2*H*-chromene-3-carbaldehyde derivative was recovered as well as some degradation products. The amount of DMSO used was optimized to a minimum, to allow an excellent stirring. The best reaction temperature was established at 130 °C, to avoid the loss of the formyl group. The amount of indole **3** added was established to prevent mixtures of hybrids and maximize the yield of the two indole unit hybrid. This careful optimization of the reaction conditions allowed us to obtain a single compound (Scheme 2) in very good yields (60 to 84%).

The MS spectrum of the obtained compound suggested that it should contain two indole units. Furthermore, the NMR experiments confirmed this hypothesis and allowed us to establish that the obtained compounds were 3,3′-[(2-aryl-4-chloro-2*H*-chromen-3-yl)methylene]bis(1*H*-indoles) **4a**–**c** as depicted in Scheme 2. We believe that the indole carbon C-3 nucleophilicity is high and that the formyl carbon electrophilicity favors the reaction with a second indole unit. The main NMR features are: (i) N*H* proton resonances at δ = 10.32–10.61 and 11.06–11.16 ppm, appearing as doublets (*J* ≈ 2 Hz) due to their coupling with H-2 in each indole unit; (ii) methinic proton of the carbon linked to the two indole units, a singlet at δ = 6.14–6.25 ppm.

**Scheme 3:**
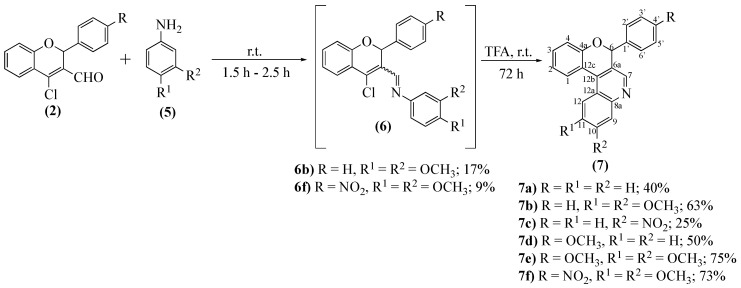
Synthesis of 6-aryl-6*H*-chromeno[3,4-*c*]quinolines **7a**–**f**.

Next, the reactivity of 2-aryl-4-chloro-2*H*-chromene-3-carbaldehydes **2a**–**c** with anilines **5** was studied. The main objective was to obtain quinoline derivatives by forming an imine intermediate, followed by its cyclization with the elimination of hydrochloric acid (Scheme 3).

The first attempts, reacting 4-chloro-2-phenyl-2*H*-chromene-3-carbaldehyde **2a** with aniline, gave a mixture of products and indications that the imine derivative was either not stable, or not able to resist the purification steps. Therefore, we decided to control the reaction by TLC, adding TFA to promote the in situ cyclization immediately after the consumption of the **2a**. This procedure proved efficient and allowed the synthesis of the desired 6-phenyl-6*H*-chromeno[3,4-*c*]quinoline **7a** in moderate yield. Naturally, the reaction scope was further evaluated (Scheme 3). Although the reaction occurs with certain success, we notice that the first step requires variable reaction times (from 1.5 to 2.5 h), whereas the second step was accomplished in an additional 72 h. Other relevant results include the lower or nonexistent reactivity of derivatives having nitro groups as substituents; only two novel compounds, **7c** and **7f**, were obtained, the former in low yield (25%). On the other hand, the presence of activating groups in the chromene-3-carbaldehydes, such as OCH_3_, improve the yield, cases of **7d** (50%), and **7e** (75%). That is more evident when two methoxy substituents are present in aniline, which increased reactivity, and all derivatives were obtained in very good yields (**7b**, 63%; **7e**, 75%, **7f**, 73%). We were also able to isolate two imine derivatives (Scheme 3) in low yields, and **6f** was obtained only when the second step was shortened 48 h.

The main features in the NMR characterization of 6-aryl-6*H*-chromeno[3,4-*c*]quinolines **7a**–**f** are the two singlets at δ = 6.32–6.44 and 7.30–7.60 ppm due to, respectively, H-6 and H-7 resonances. Another typical signal is the double doublet appearing at δ = 8.40–8.55 ppm and corresponding to the H-1 resonance. These signals were not only in agreement with the proposed structure (Scheme 3) but also showed essential connectivities in the HMBC spectra that allowed the full characterization of the synthesized 6-aryl-6*H*-chromeno[3,4-*c*]quinolines **7a**–**f**.

**Scheme 4:**
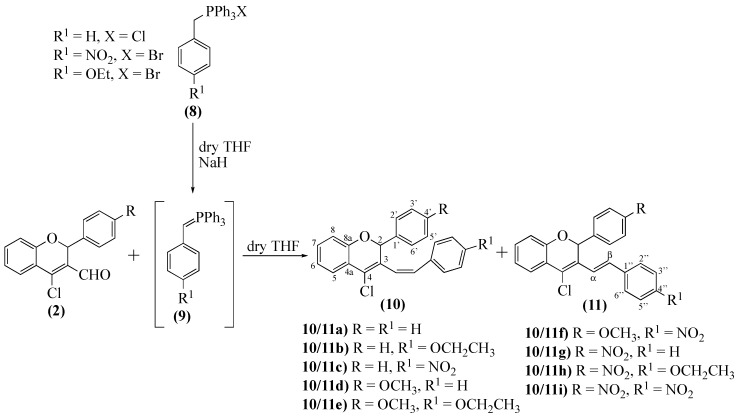
Synthesis of (*Z*- and *E*)-2-aryl-4-chloro-3-styryl-2*H*-chromenes **10a**–**i and 11a**–**i**.

3-Styrylchromenes constitute a small group of synthetic compounds recently reported for their antiviral [3], cytotoxic [28], and antimicrotubular activity [29]. Several methods for the synthesis of the 3-styryl-*2H*-chromene nucleus have been described, including the Horner-Wadsworth-Emmons [3] and Wittig [29] reactions. Considering this, we decided to assess the reactivity of 2-aryl-4-chloro-2*H*-chromene-3-carbaldehydes **2a**–**c** in a Wittig reaction with aromatic ylides **9** (Scheme 4). Our first approach was to use a previously established procedure [30], although with some adaptations, mainly in the reaction times and the amount of base. The two isomers were always obtained, with a clear preference for (*Z*)-2-aryl-4-chloro-3-styryl-2*H*-chromenes **11a**–**f** (Table 1, Scheme 4), as predicted by Wittig reaction selectivity [31].

The optimization steps demonstrated that the Wittig reaction results are dependent on both the aldehyde **2** and the ylide **9**. It is clear that the ylide **9c**, having an electron-donating group, requires less time to be formed, depends on the amount of base used and, in our opinion, also on the solvent dryness; freshly distilled THF holds better results (Table 1). The aldehyde **2a** was less reactive. It requires longer reaction times, and the major isomer is obtained in lower yields (Table 1). Another interesting result is that aldehyde **2c** presents better results if the second step is performed at room temperature (Table 1); in fact, reflux promotes aldehyde decomposition instead of improving its reactivity.

The most important features of the ^1^H-NMR spectra of 2-aryl-4-chloro-3-styryl-2*H*-chromenes **10a**–**i** and **11a**–**i** are the signals of the vinylic protons, which allowed the establishment of the system absolute configuration. In the (*Z*)-isomer **10a**–**i**, the resonance of H-β (6.56-6.74 ppm) appears at higher frequency values than those of H-α (6.22–6.63 ppm), and the stereochemistry of the vinylic system was established as (*Z*) due to typical coupling constant values (^3^*J*_H-α-H-β_ 12.1–12.3 Hz). In contrast, in the (*E*)-isomer **11a**–**i**, is the resonance of H-α (7.42–7.75 ppm) that appears at higher frequency values, due to the chlorine atom’s proximity, than those of H-β (6.42–6.50 ppm). The vinylic system’s stereochemistry was established as (*E*) due to typical coupling constant values (^3^*J*_H-α-H-β_ 16.5–16.6 Hz) and confirmed by the NOESY experiments.

**Scheme 5:**
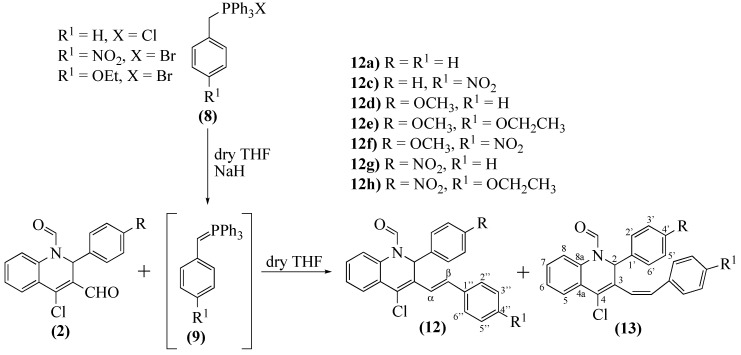
Synthesis of (*Z*- and *E*)-2-aryl-4-chloro-3-styrylquinoline-1(2*H*)-carbaldehydes **12a**–**i** and **13a**–**i**.

The success achieved in the synthesis of 2-aryl-4-chloro-3-styryl-2*H*-chromene derivatives prompt us to use the 2-aryl-4-chloro-1,2-dihydroquinoline-1,3-dicarbaldehydes **2d**–**f**, aiming to obtain new (*Z*- and *E*)-2-aryl-4-chloro-3-styrylquinolines. Therefore, we considered a previously established procedure for the synthesis of 3-styryl-4-quinolones [32], using the reaction of 2-aryl-4-chloro-1,2-dihydroquinoline-1,3-dicarbaldehydes **2d**–**f** with ylides **9a**–**c** formed in situ from the phosphonium salts **8a**–**c** (Scheme 5). A color change and TLC analysis controlled the formation of ylide, being the ideal time for the ylides formation 15 min, 3 min, and 7 min, respectively, for **9a**, **9b**, and **9c**. After the ylide formation, aldehydes **2d**–**f** were added to the reaction mixture. The first results were not as good as expected (Table 2; Entries 1, 8, and 15). As so, the reaction conditions were individually studied (Table 2). The analysis of Table 2 shows that the results depend on the aldehyde’s reactivity and that the second step’s reaction time is a crucial parameter for the transformation.

Another significant observation is that in this reaction, the (*E*)-isomer was the major product. In contrast, the (*Z*)-isomer was obtained in vestigial amounts or just detected as an impurity in the ^1^H-NMR spectra. For this reason, none of the (*Z*)-isomers were obtained as pure compounds. In a few cases (Table 2; Entries 4, 5, 18, and 19), we could not isolate the (*E*)-isomer, because they are light-sensitive, especially when in solution or silica gel. In other cases, increasing the second step reaction time allowed the yield improvement (Table 2; Entries 3, 7, 14, and 16).

The ylide’s reactivity seems to influence the result, with the ylide **9c** (R^1^=NO_2_) as the less reactive, probably due to the electron-withdrawing group. Although **9b** (R^1^=OCH_2_CH_3_) appeared to be more reactive, with shorter reaction times in a few examples (Table 2; Entries 11 and 17), the yields were not as good as expected. In terms of the aldehyde’s reactivity, 2-aryl-4-chloro-1,2-dihydroquinoline-1,3-dicarbaldehyde **2e** is the most reactive. We were able to obtain all (*E*)-isomers, some in moderate yields (Table 2; Entries 8 and 14). Three crucial conclusions can be reached from these results:

(i) 2-Aryl-4-chloro-1,2-dihydroquinoline-1,3-dicarbaldehydes **2d**–**f** are not as reactive as the 2-aryl-4-chloro-3-styryl-2*H*-chromenes **2a**–**c**;
(ii) The common Wittig reaction stereoselectivity was not observed, the (*E*)-isomer was obtained;
(iii) The formyl group at C-1 did not undergo Wittig reaction, naturally because of the amide carbonyl group’s lower nucleophilicity.

The main features in the ^1^H-NMR spectra of (*E*)-2-aryl-4-chloro-3-styrylquinoline-1(2*H*)-carbaldehydes **12a**–**i** are the resonances of *H*-α and *H*-β, which appear as two doublets at δ = 7.40–7.74 and 6.69–6.81 ppm, respectively, and have coupling constants (*J* ≈ 16 Hz) typical of an (*E*)-configuration. The depicted 3-styryl group conformation (Scheme 5) was confirmed by NOESY experiments in whicH-NOE correlations between *H*-β and *H*-2, *H*-2′ were observed.

A DFT analysis on the reaction of benzyl triphenylphosphonium ylide with **2a** was performed to rationalize the abnormal selectivity obtained with the used complex aldehydes, considering the possible formation of both (*E* and *Z*)-isomers. Previous reports have shown that the Wittig reaction mechanism witH-Non-stabilized and semi-stabilized ylides proceeds through an early four-centre transition state leading to the irreversible formation of a phosphoxetane intermediate; evidence clearly discards the alternative existence of a betaine species. The reaction then passes through a secondary transition state as the phosphoxetane degenerates to the desired alkene product. It has been established in the literature that this first transition state’s nature determines the stereochemistry of the reaction, locking the relative position of the aldehyde and ylide irreversibly [31].

**Scheme 6:**
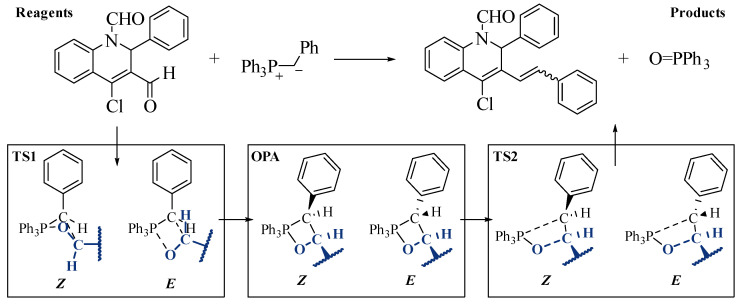
Preliminary investigation into the mechanism of the Wittig reaction.

Preliminary studies using the low level of theory B3LYP/STO-3G* to investigate the Wittig reaction mechanism for these substrates were performed. Indeed, we verified the existence of a first high-energy transition state (TS1), leading to planar OPA intermediates (Scheme 6). Significant puckering was observed in TS1 for the (*Z*)-isomer, reducing unfavourable interactions between the aldehyde and ylide substituents. The latter formation of the alkene undergoes a second transition state (TS2) with the P-C bond’s advanced cleavage. Here too, some puckering is observed. Most importantly, this secondary transition state was much lower in energy than TS1, supporting our hypothesis that selectivity is controlled by relative configuration at TS1 and that the reaction is irreversible after this point.

Graph 1 shows the calculated potential energy profile for the reaction in study, here using a more precise 6-31G(d) basis set. A similar high-energy transition state preceding phosphoxetane formation was found for both configurations. The transition state TS1, shown in Scheme 6, has a geometry consistent with previous works, with advanced formation of the C-C bond (1.94–2.14 Â) but mainly electrostatic P-O interaction over 3 Â. The energy barrier of this rate-determining step of over 10 kcal/mol is higher than expected. This is most likely due to the complexity of the aldehyde structure, increasing steric constraints in the transition state, and the charge distribution to the conjugated heterocyclic ring.

**Figure 1:**
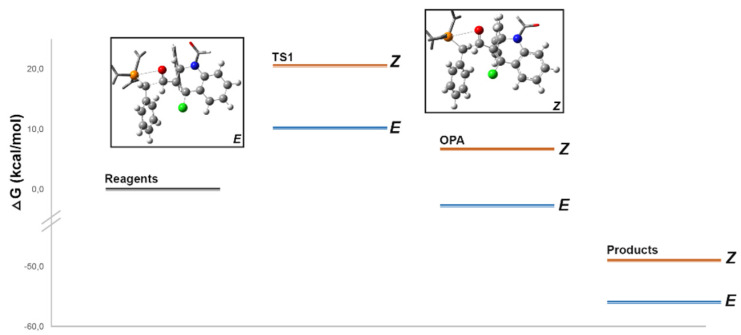
Calculated potential energy surface for the reaction of **2a** with triphenylphosphonium ylide **9a**.

Comparing both isomers (Figure 1), significantly lower energy was found for the TS leading to the (*E*)-isomer, in clear contradiction with most experimental and computational studies on semi-stabilized ylides but in agreement with our experimental results. This suggests that additional factors may play a role. In both cases, the transition state has an early character (C-C bond ~2.1 Â) that reduces steric interactions between substituents.

Furthermore, the non-aromatiC-Nature of the aldehyde substituent allows increased flexibility to reduce any steric constraints. However, the presence of the large aryl substituent on the C-2 sp^3^ carbon limits its ability to assume a puckered state (described as an increase in the PCCO dihedral angle) as it approaches the phosphine phenyl substituents. This is particularly relevant in the (*Z*)-isomer, where puckering acts as a tool to reduce unfavorable interactions between the aldehyde and both the phosphine and ylide substituents and thus reduce the energy of the transition state. Therefore, the restricted puckering of the (*Z*)-TS1 may explain its higher energy in our calculations and the preference for the formation of the *E* quinoline product.

## 3. Materials and Methods

### 3.1. General Experimental Procedures

Melting points were determined on a B-545 melting point apparatus (Büchi, Porto, Portugal) and are uncorrected. ^1^H and ^13^C-NMR spectra were recorded on Avance III 300, Avance III HD 500, and Avance III HD 700 [300.13 MHz (^1^H), 75.47 MHz (^13^C); 500.13 MHz (^1^H), 125.76 MHz (^13^C); 700.13 MHz (^1^H), 176,05 MHz (^13^C)] spectrometers (Bruker, Wissembourg, France) with TMS as internal reference and with CDCl_3_ or DMSO*d*_6_ as solvent. Chemical shifts (δ) are reported in parts per million values and coupling constants (*J*) in Hertz. Unequivocal ^1^H assignments were made using 2D NOESY experiments (mixing time of 800 ms), while ^13^C assignments were made based on 2D gHSQC (^1^H/^13^C) and gHMBC (delays for one bond and long-range J_C/H_ couplings were optimized for 145 and 7 Hz, respectively) experiments. Positive-ion ESI mass spectra were acquired using a Q-TOF2 instrument (Bruker, Wissembourg, France). Nitrogen was used as nebulizer gas and argon as collision gas. The needle voltage was set at 3000 V, with the ion source at 80 °C and desolvation temperature at 150 °C. Cone voltage was 35 V. High resolution mass spectra (HRMS-ESI^+^) were measure in a microTOF (focus) mass spectrometer. Ions were generated using an Apollo ll (ESI) source (Bruker, Wissembourg, France). Ionization was achieved by electrospray, using a voltage of 4500 V applied to the needle and a counter voltage between 100 and 150 V applied to the capillary. Elemental analyses were obtained with a 932 CHNS analyser (LECO, Madrid, Spain). Preparative thin layer chromatography was carried out with Silica gel 60 GF_254_ (Merck KGaA, Porto, Portugal) and column chromatography using Silica gel 0.060–0.020 mm 60A (Acros Organics, Porto, Portugal). All other chemicals and solvents were obtained from commercial sources and used as received or dried using standard procedures.

### 3.2. General Procedure for the Vilsmeyer-Haack Formylation of Flavanones ***1a**–**c*** and 2-aryl-2,3-dihydroquinolin-4(1H)-one ***1d**–**f***

A mixture of POCl_3_ (0.1 mL, 1 mmol) in dry DMF (2 mL) was stirred under 0 °C during 15 min. After that period, a solution of the appropriate 2-aryl-2,3-dihydroquinolin-4(1*H*)one **1d**–**f** (0.1 mmol in 10 mL CH_2_Cl_2_) or the appropriate flavanone **1a**–**c** (21 mg, 0.1 mmol) was added and the reaction was stirred at ~60 °C for 1.5 h to 2 h (in the case of 2-aryl-2,3-dihydroquinolin-4(1*H*)one **1d**–**f**) or 10 to 12 h (flavanone **1a**–**c**), depending on the starting material consumption. Then it was poured into a mixture of ice/water (10 g/15 mL) and the pH value was adjusted to 5 using a saturated solution of NaHCO_3_. The solid obtained was filtered, dissolved in dichloromethane (25 mL) and the organic phase was washed with water (3 × 25 mL). The organic phase was dried over anhydrous sodium sulfate and evaporated. The residue was purified by preparative thin layer chromatography using appropriate mixtures of eluent, CH_2_Cl_2_/hexane.

4-Chloro-2-phenyl-2*H*-chromene-3-carbaldehyde (**2a**). Yellow solid, yield (η) 95%, m. p. 101–103 °C. ^1^H-NMR (300.13 MHz, CDCl_3_): δ 6.40 (s, 1H, H-2); 6.89 (dd, 1H, H-8, *J* 1.3 and 8.1 Hz); 7.03 (ddd, 1H, H-6, *J* 1.3, 7.1 and 8.1 Hz); 7.25-7.31 (m, 5H, H-2′,3′,4′,5′,6′); 7.35 (ddd, 1H, H-7, *J* 1.3, 7.1 and 8.1 Hz); 7.72 (dd, 1H, H-5, *J* 1.3 and 8.1 Hz); 10.30 (s, 1H, 3-CHO) ppm. ^13^C-NMR (75.47 MHz; CDCl_3_): δ 75.0 (C-2); 117.4 (C-8); 120.0 (C-4a); 122.0 (C-6); 126.6 (C-5); 126.8 (C-2′,6′); 128.5 (C-3′,5′); 128.8 (C-4′); 134.5 (C-7); 138.1 (C-1′), 143.9 (C-4); 155.1 (C-8a); 188.2 (3-CHO) ppm. ESI^+^-MS *m/z* (%): 271.1 ^35^Cl [M *+* H]^+^ (36); 273.1 ^37^Cl [M + H]^+^ (24); 293.1 ^35^Cl [M *+* Na]^+^ (70); 295.1 ^37^Cl [M + Na]^+^ (6). EI-HRMS: calcd for (C_16_H_11_O_2_^35^Cl) 270.0448; found 270.0441. Calcd for (C_16_H_11_O_2_^37^Cl) 272.0418; found 272.0409.

4-Chloro-2-(4-methoxyphenyl)-2*H*-chromene-3-carbaldehyde (**2b**). Yellow solid, η 77%, m. p. 78–80 °C. ^1^H-NMR (300.13 MHz, CDCl_3_): δ 3.74 (s, 3H, 4′-OCH_3_); 6.33 (s,1H, H-2); 6.79 (d large, 2H, H-3′,5′, *J* 9.2 Hz); 6,85 (dd, 1H, H-8, *J* 1.3 and 8.0 Hz); 7.02 (ddd, 1H, H-6, *J* 1.3, 7.1 and 8.0 Hz); 7.22 (dlarge, 2H, H-2′,6′, *J* 9.2 Hz); 7.33 (ddd, 1H, H-7, *J* 1.3, 7.1 and 8.0 Hz); 7.73 (dd, 1H, H-5, *J* 1.3 and 8.0 Hz); 10.27 (s, 1H, 3-CHO) ppm. ^13^C-NMR (75.47 MHz; CDCl_3_): δ 55.2 (4′-OCH_3_); 74.9 (C-2); 113.9 (C-3′,5′); 117.5 (C-8); 120.0 (C-4a); 121.9 (C-6); 126.4 (C-5); 126.9 (C-3); 128.3 (C-2′,6′); 130.1 (C-1′); 134.4 (C-7); 143.6 (C-4′); 143.9 (C-4); 155.0 (C-8a); 188.1 (3-CHO) ppm. ESI^+^-MS *m/z* (%): 301.1 ^35^Cl [M + H]^+^ (5); 303.1 ^37^Cl [M + H]^+^ (100); 323.1 ^35^Cl [M + Na]^+^ (40); 325.1 ^37^Cl [M + Na]^+^ (2). EI^+^-HRMS: calcd for (C^17^H_13_O_3_^35^Cl) 300.0553, found 300.0563. Calcd. for (C_17_H_13_O_3_^37^Cl) 302.0524, found 302.0527.

4-Chloro-2-(4-nitrophenyl)-2*H*-chromene-3-carbaldehyde (**2c**). Yellow solid, η 66%, m. p. 187–188 °C. ^1^H-NMR (300.13 MHz, CDCl_3_): δ 6.47 (s, 1H, H-2); 6.97 (dd, 1H, H-8, *J* 1.3 and 8.1 Hz); 7.08 (ddd, 1H, H-6, *J* 1.3, 7.1 and 8.1 Hz); 7.42 (ddd, 1H, H-7, *J* 1.3, 7.1 and 8.1 Hz); 7.47 (dlargo, 2H, H-2′,6′, *J* 9.0 Hz); 7.74 (dd, 1H, H-5, *J* 1.3 and 8.1 Hz); 8.13 (dlargo, 2H, H-3′,5′, *J* 9.0 Hz); 10.32 (s, 1H, 3-CHO) ppm. ^13^C-NMR (75.47 MHz; CDCl_3_): δ 73.8 (C-2); 117.4 (C-8); 119.0 (C-4a); 122.7 (C-6); 123.8 (C-3′,5′); 126.0 (C-3); 126.9 (C-5); 127.6 (C-2′,6′), 135.1 (C-7); 144.5 (C-4); 145.6 (C-1′); 147.2 (C-4′); 154.6 (C-8a); 188.1 (3-CHO) ppm. ESI^+^-MS *m/z* (%): 316.1 ^35^Cl [M + H]^+^ (100); 318.1 ^37^Cl [M + H]^+^ (40); 338.1 ^35^Cl [M + Na]^+^ (29); 340.1 ^37^Cl [M + Na]^+^ (3). EI^+^-HRMS: calcd for (C_16_H_10_NO_4_^35^Cl) 315.0298, found 315.0304. Calcd for (C_16_H_10_NO_4_^37^Cl) 317.0269, found 317.0278.

4-Chloro-2-phenyl-1,2-dihydroquinoline-1,3-dicarbaldehyde (**2d**). Yellow oil, η 60%. ^1^H-NMR (300.13 MHz; CDCl_3_): δ 6.94 (s, 1H, H-2); 7.09-7.20 (m, 6H, H-2′,3′,4′,5′,6′,8); 7.36 (t, 1H, H-6, *J* 7.6 Hz); 7.49 (t, 1H, H-7, *J* 7.6 Hz); 8.00 (d, 1H, H-5, *J* 7.8 Hz); 8.70 (s, 1H, NCHO); 10.32 (s, 1H, 3-CHO) ppm. ^13^C-NMR (75.47 MHz; CDCl3): δ 50.6 (C-2); 118.4 (C-8); 126.0 (C-6); 127.1 (C-3′,4′); 127.7 (C-5); 128.5 (C-4′); 128.7 (C-2′,6′); 131.0 (C-3); 133.2 (C-1′); 133.4 (C-7); 136.8 (C-4a); 137.2 (C-8a); 142.9 (C-4); 160.6 (1-NCHO); 187.8 (3-CHO) ppm. MS (ESI^+^) *m/z* (%): 320.0 ^35^Cl [M *+* Na]^+^ (100); 322.0 ^37^Cl [M *+* Na]^+^ (3).

4-Chloro-2-(4-methoxyphenyl)-1,2-dihydroquinoline-1,3-dicarbaldehyde (**2e**). Yellow oil, η 70%. ^1^H-NMR (300.13 MHz; CDCl_3_): δ 3.71(s, 3H, 4′-OCH_3_); 6.72 (d, 2H, H-3′,5′, *J* 8.6 Hz); 6.88 (s, 1H, H-2); 7.12 (d, 2H, H-2′,6′, *J* 8.6 Hz); 7.11-7.15 (m, 1H, H-8); 7.36 (t, 1H, H-6, *J* 7.7 Hz); 7.48 (t, 1H, H-7, *J* 7.7 Hz); 8.00 (d, 1H, H-5, *J* 7.8 Hz); 8.69 (s, 1H, NCHO); 10.30 (s, 1H, CHO) ppm. ^13^C-NMR (75.47 MHz; CDCl_3_): δ 50.2 (C-2); 55.2 (4′-OCH_3_); 114.0 (C-3′,5′); 118.4 (C-8); 126.0 (C-6); 127.4 (C-4a); 127.6 (C-5); 128.5 (C-2′,6′); 129.5 (C-1′); 131.7 (C-3); 133.4 (C-7); 136.7 (C-8a); 142.8 (C-4); 159.6 (C-4′); 160.5 (1-NCHO); 187.8 (3-CHO) ppm. MS (ESI^+^) *m/z* (%): 350.1 ^35^Cl [M *+* Na]^+^ (100); 352,1 ^37^Cl [M *+* Na]^+^ (40).

4-Chloro-2-(4-nitrophenyl)-1,2-dihydroquinoline-1,3-dicarbaldehyde (**2f**). Orange solid, η 40%, m.p. 151–153 °C. ^1^H-NMR (300.13 MHz; CDCl_3_): δ 7.00 (s, 1H, H-2); 7.19 (d, 1H, H-8, *J* 7.9 Hz); 7.37 (d, 2H, H-2′,6′, *J* 8.8 Hz); 7.42 (t, 1H, H-6, *J* 7.6 Hz); 7.54 (t, 1H, H-7, *J* 7.6 Hz); 8.03 (d, 1H, H-5, *J* 7.9 Hz); 8.07 (d, 2H, H-3′,5′, *J* 8.8 Hz); 8.72 (s, 1H, 1-NCHO); 10.33 (s, 1H, 3-CHO) ppm. ^13^C-NMR (75.47 MHz; CDCl_3_): δ 50.5 (C-2); 118.4 (C-8); 123.8 (C-4a); 124.0 (C-3′,5′); 126.5 (C-6); 128.0 (C-5); 128.1 (C-2′,6′); 130.0 (C-3); 134.0 (C-7); 136.3 (C-8a); 144.0 (C-4); 144.1 (C-1′); 147.8 (C-4′); 160.6 (1-NCHO); 187.6 (3-CHO) ppm. MS (ESI^+^) *m/z* (%): 343.1 ^35^Cl [M *+* H]^+^ (15); 345.1 ^37^Cl [M *+* H]^+^ (2); 381.3 ^35^Cl [M *+* K]^+^ (100); 383,0 ^37^Cl [M *+* K]^+^ (15). EI-HRMS: calcd. for (C_17_H_11_N_2_O_4_^35^Cl) 342.0407; found 342.0415. Calcd for (C_17_H_11_N_2_O_4_^37^Cl) 344.0378; found 344.0391.

### 3.3. General Procedures for the Synthesis of the Chromene-Indole Hybrids ***4a**–**c***

An excess of indole **3** (94 mg, 0.80 mmol) was added to a round bottom flask along with the appropriate 2-aryl-4-chloro-2*H*-chromene-3-carbaldehyde **2a**–**c** (0.26 mmol) and dissolved in DMSO (2.5 mL). The mixture was stirred and heated to 130 °C during 24 h. At the end, the reaction mixture was poured into a mixture of ice/water (10 g/20 mL) and liquid-liquid extraction was performed with dichloromethane (3 × 30 mL). The organic layer dried over anhydrous sodium sulfate, solvent evaporated and the residue purified by thin layer chromatography using CH_2_Cl_2_ as eluent.

3,3′-[(4-Chloro-2-phenyl-2*H*-chromen-3-yl)methylene]bis(1*H*-indole) (**4a**). Yellow solid, η 79%, m.p. 106-107 °C. ^1^H-NMR (500.13 MHz; CDCl_3_): δ 5.94 (s, 1H, H-2″); 6.25 (s, 1H, 3″-C*H*CroInd); 6.41 (d, 1H, H-2, *J* 2.0 Hz); 6.65–6.69 (m, 3H, H-8″, 3″′, 5″′); 6.73–6.80 (m, 3H, H-2″′, 4″′, 6″′); 6.92–7.03 (m, 5H, H-6″, 5, 6, 7, 5′); 7.89 (dt, 1H, H-7″, *J* 1.6 and 7.8 Hz); 7.17 (dt, 1H, H-6′, *J* 1.0 and 7.9 Hz); 7.25–7.29 (m, 1H, H-4′); 7.37 (d, 1H, H-7′, *J* 7.9 Hz); 7.50 (d, 1H, H-2′, *J* 1.5 Hz); 7.53–7.56 (m, 1H, H-4); 7.64 (dd, 1H, H-5″, *J* 1.6 and 7.8 Hz); 10.32 (d, 1H, H-1); 11.10 (large s, 1H, H-1′) ppm. ^13^C-NMR (125.77 MHz; CDCl_3_): δ 36.2 (3″-*C*HCroInd); 79.0 (C-2″); 110.6 (C-7); 111.2 (C-7′); 114.0 (C-3); 115.7 (C-3′); 116.7 (C-8″); 119.2 (2C, C-5, 5′); 119.3 (C-4); 120.6 (C-4′); 121.5 (C-6); 121.8 (C-6″); 122.2 (C-6′); 122.8 (C-2′); 123.1 (C-4″a); 123.7 (C-2); 124.8 (C-5″); 125.0 (C-4″); 126.9 (C-3′a); 127.2 (C-3″′, 5″′, 3a); 127.4 (C-2″′, 6″′); 127.6 (C-4″′); 130.0 (C-7″); 132.1 (C-3″); 135.9 (C-7a); 136.6 (C-1″′); 136.9 (C-7′a); 152.4 (C-8″a) ppm. ESI^+^-MS *m/z* (%): 509.2 ^35^Cl [M *+* Na]^+^ (100); 511.2 ^37^Cl [M *+* Na]^+^ (25). EI-HRMS: calcd. for (C_32_H_23_N_2_O^35^Cl) 486.1499; found 486.1490. Calcd for (C_32_H_23_N_2_O^37^Cl) 488.1470; found 488.1465.

3,3′-[(4-chloro-2-(4-methoxyphenyl)-2*H*-chromen-3-yl)methylene]bis(1*H*-indole) (**4b**). red solid, η 84%, m.p. 119–121 °C. ^1^H-NMR (500.13 MHz, DMSO-D_6_): δ 3.50 (s, 3H, 4″′-OC*H*_3_); 6.01 (s, 1H, H-2″); 6.15 (s, 1H, 3″-C*H*CroInd); 6.30 (d, 2H, H-3″′, 5″′, *J* 8.6 Hz); 6.65 (d, 1H, H-2, *J* 2.0 Hz); 6.65–6.67 (m, 1H, H-8″); 6.71 (d, 2H, H-2″′, 6″′, *J* 8.6 Hz); 6.87 (t, 1H, H-5′, *J* 7.5 Hz); 6.89–7.01 (m, 3H, H- 6″, 5, 6); 7.04–7.10 (m, 2H, H-7, 6′); 7.13 (dt, 1H, H- 7″, *J* 1.0 and 7.7 Hz); 7.24 (d, 1H, H-4′, *J* 8.0 Hz); 7.35 (d, 1H, H-2′, *J* 1.5 Hz); 7.41 (d, 1H, H-7′, *J* 8.1 Hz); 7.45 (d, 1H, H-4, *J* 8.1 Hz); 7.54 (dd, 1H, H-5″, *J* 1.0 and 7.7 Hz); 10.57 (d, 1H, H-1, *J* 2.0 Hz); 11.06 (d, 1H, H-1′, *J* 1.5 Hz) ppm. ^13^C-NMR (125.77 MHz, DMSO-*d*_6_): δ 35.6 (3″-*C*HCroInd); 54.8 (4″′-O*C*H_3_); 77.5 (C-2″); 111.1 (C-7); 111.6 (C-7′); 112.0 (C-3) 112.5 (C-3″′, 5″′); 114.3 (C- 3′); 116.5 (C-8″); 118.2 (C-4′); 118.2 (C-5); 118.4 (C-5′); 119.0 (C-4); 120.7 (C-6); 121.2 (C-6′); 121.6 (C-6″); 122.6 (C-4″); 122.7 (C-4″a); 123.2 (C-2′); 124.3 (C-5″, 2); 126.5 (C-3′a); 127.0 (C-3a); 128.6 (C-2″′, 6″′); 128.8 (C-1″′); 130.1 (C-7″); 133.6 (C-3″); 135.8 (C-7a); 136.6 (C-7′a); 151.8 (C-8″a); 158.6 (C-4″′) ppm. ESI^+^-MS *m/z* (%): 539.2 ^35^Cl [M *+* Na]^+^ (29); 541.2 ^37^Cl [M *+* Na]^+^ (12). EI-HRMS: calcd. for (C_33_H_25_N_2_O_2_^35^Cl) 516.1605; found 516.1610. Calcd for (C_33_H_25_N_2_O_2_^37^Cl) 518.1575; found 518.1577.

3,3′-[(4-Chloro-2-(4-nitrophenyl)-2*H*-chromen-3-yl)methylene]bis(1*H*-indole) (**4c**). Yellow solid, η 60%, m.p. 179–180 °C. ^1^H-NMR (500.13 MHz, DMSO-*d*_6_): δ 6.14 (s, 1H, 3″-C*H*CroInd); 6.26 (s, 1H, H-2″); 6.69 (dd, 1H, H- 8″, *J* 0.8 and 8.0 Hz); 6.75 (d, 1H, H-2, *J* 2.1 Hz); 6.82–6.94 (m, 6H, H-2″′, 6″′, 5, 6, 7, 5′); 7.03–7.11 (m, 2H, H-6″, 6′); 7.17 (dt, 1H, H-7″, *J* 1.6 and 8.0 Hz); 7.27 (d, 1H, H-4′, *J* 8.0 Hz); 7.34 (d, 1H, H-4, *J* 7.4 Hz); 7.41–7.46 (m, 3H, H-3″′, 5″′, 7′); 7.56 (d, 1H, H-2′, *J* 1.6 Hz); 7.62 (dd, 1H, H-5″, *J* 1.6 and 7.8 Hz); 10.61 (d, 1H, H-1, *J* 2.1 Hz); 11.16 (d, 1H, H-1′, *J* 1.6 Hz) ppm. ^13^C-NMR (125.77 MHz, DMSO-*d*_6_): δ 35.7 (3″-*C*HCroInd); 77.1 (C-2″); 111.4 (C-7); 112.1 (C-3); 112.1 (C-7′); 113.8 (C-3′); 116.9 (C-8″); 118.6 (C-4); 118.9 (C-5, 5′); 119.6 (C-4′); 121.3 (C-6); 121.7 (C-6′); 122.2 (C-3″′, 5″′); 122.4 (C-6″); 122.6 (C-4″a); 123.3 (C-2′); 123.8 (C-4″); 125.0 (C-5″); 125.2 (C-2); 126.9 (C-3′a); 127.1 (C-3a); 128.3 (C-2″′, 6″′); 131.0 (C-7″); 133.5 (C-3″); 136.2 (C-7a); 137.0 (C-7′a); 143.2 (C-1″′); 146.4 (C-4″′); 151.7 (C-8″a) ppm. ESI^+^-MS *m/z* (%): 554.2 ^35^Cl [M *+* Na]^+^ (8); 556.2 ^37^Cl [M *+* Na]^+^ (3). EI-HRMS: calcd. for (C_32_H_22_N_3_O_3_^35^Cl) 531.1350; found 531.1354. Calcd for (C_32_H_22_N_3_O_3_^37^Cl) 533.1320; found 533.1327.

### 3.4. General Procedures for the Synthesis of 6-Aryl-6H-chromeno[3,4-c]quinolines ***7a**–**f***

In a round bottom flask with solution of the appropriate 2-aryl-4-chloro-2*H*-chromene-3-carbaldehyde **2a**–**c** (0.10 mmol) in dichloromethane (7 mL) was added the correspondent aniline **5** (0.27 mmol). The mixture was stirred at room temperature for 1.5 or 2.5 h depending on the substituents in the 2-aryl-4-chloro-2*H*-chromene-3-carbaldehydes **2a**–**c**. After this period, TFA (2.6 mmol in case of 3,4-dimethoxyanilinine and 26 mmol in case of aniline or 4-nitroaniline) was added to the reaction mixture and left stirring at room temperature for 72 h. Finally, the solvent was evaporated on the rotary evaporator and the mixture was purified by preparative chromatography using CH_2_Cl_2_ or a mixture of CH_2_Cl_2_/hexane (3:1) as eluent.

1-[4-Chloro-2-phenyl-2*H*-chromen-3-yl)]-*N*-(3,4-dimethoxyphenyl)methanimine (**6b**). Yellow solid, η 17%, m.p. 79–81 °C. ^1^H-NMR (500.13 MHz, CDCl_3_): δ 3.90 and 3.91 (s, 3H, 3″- and 4″-OC*H*_3_); 6.79 (s, 1H, H-2), 6.81 (dd, 1H, H-6″, *J* 2.3 and 6.0 Hz); 6.83 (d, 1H, H- 2″, *J* 2.3 Hz); 6.86 (d, 1H, H-5″, *J* 6.0 Hz); 6.90 (dd, 1H, H-8, *J* 1.1 and 8.1 Hz); 6.99 (dt, 1H, H-6, *J* 1.0 and 7.8 Hz); 7.41 (d, 2H, H-2′,6′, *J* 8.8 Hz); 7.21–7.28 (m, 4H, H-7,3′,4′,5′); 7.63 (dd, 1H, H-5, *J* 1.5 and 7.8 Hz); 8.84 (s, 1H, C*H*N) ppm. ^13^C-NMR (125.77 MHz, CDCl_3_): δ 56.0 and 56.1 (3″- and 4″-O*C*H_3_); 76.0 (C-2); 105.7 (C-6″); 111.2 (C-5″); 112.9 (C-2″); 117.2 (C-8); 121.5 (C- 4a); 121.7 (C-6); 125.6 (C-5); 127.3 (C-2′,6′); 127.9 (C-3); 128.3 (C-3′,4′,5′); 132.2 (C-7); 134.8 (C-4); 138.8 (C-1′); 144.7 (C-1″); 148.4 (C-4″); 149.3 (C-3″); 153.0 (*C*HN); 154.2 (C-8a) ppm. ESI^+^-MS *m/z* (%): 406.1 ^35^Cl [M *+* H]^+^ (100); 408.1 ^37^Cl [M *+* H]^+^ (52).

1-[4-Chloro-2-(4-nitrophenyl)-2*H*-chromen-3-yl)]-*N*-(3,4-dimethoxyphenyl)methanimine (**6f**). Yellow solid, η 9%, m.p. 109–112 °C. ^1^H-NMR (300.13 MHz, CDCl_3_): δ 3.91 and 3.92 (s, 3H, 3″- and 4″-OC*H*_3_); 6.81 (t, 1H, H-2″, *J* 2.0 Hz); 6.84–6.87 (m, 3H, H-2,6″,5″); 6.95 (dd, 1H, H-8, *J* 1.1 and 7.9 Hz); 7.03 (dt, 1H, H-6, *J* 1.1 and 7.9 Hz); 7.30 (dt, 1H, H-7, *J* 1.6 and 7.9 Hz); 7.59 (d, 2H, H-2′,6′, *J* 8.8 Hz); 7.64 (dd, 1H, H-5, *J* 1.6 and 7.9 Hz); 8.11 (d, 1H, H-3′,5′, *J* 8.8 Hz); 8.87 (s, 1H, C*H*N) ppm. ^13^C-NMR (75.47 MHz, CDCl_3_): δ 56.0 and 56.1 (3″- and 4″-O*C*H_3_); 75.0 (C-2); 105.5 (C-6″); 111.3 (C-5″); 113.2 (C-2″); 117.1 (C-8); 121.1 (C- 4a); 122.3 (C-6); 123.5 (C-3′,5′); 125.9 (C-5); 127.1 (C-3); 128.1 (C-2′,6′); 132.7 (C- 7); 135.4 (C-4); 144.1 (C-1″); 146.1 (C-1′); 147.7 (C-4′); 148.8 (C-3″); 149.5 (C-4″); 152.5 (*C*HN); 153.7 (C-8a) ppm. ESI^+^-MS *m/z* (%): 451.1 ^35^Cl [M *+* H]^+^ (100); 453.1 ^37^Cl [M *+* H]^+^ (30).

6-Phenyl-6*H*-chromeno[3,4-c]quinoline (**7a**). Yellow oil, η 40%. ^1^H-NMR (500,13 MHz, CDCl_3_): δ 6.37 (s, 1H, H-6); 7.05 (dd, 1H, H-4, *J* 1.3 and 7.9 Hz); 7.18 (dt, 1H, H-2, *J* 1.3 and 7.5 Hz); 7.39 (dt, 1H, H-3, *J* 1.3 and 7.5 Hz); 7.41–7.50 (m, 7H, H-7,11, 2′,3′,4′,5′,6′); 7.66 (d, 1H, H-12, J 8.3 Hz); 7.70 (dt, 1H, H-10, J 1.4 and 7.0 Hz); 8.15 (d, 1H, H-9, J 8.3 Hz); 8.50 (dd, 1H, H-1, J 1.3 and 7.9 Hz) ppm. ^13^C-NMR (500,13 MHz, CDCl_3_): δ 80.3 (C-6); 117.7 (C-4); 122.6 (C-2); 123.2 (C-12c); 125.6 (C-1); 126.3 (C-11); 126.5 (C-6a); 127.5 (C-12a); 127.8 (C-12); 128.2 (C-2′,6′); 128.8 (C-3′,5′); 129.0 (C-4′); 129.3 (C-9); 129.8 (C-10); 132.0 (C-3); 132.9 (C-7); 138.6 (C-1′); 148.1 (C-8a); 148.9 (C-12b); 156.6 (C-4a) ppm. ESI^+^-MS *m/z* (%):310.2 [M *+* H]^+^ (42).

10,11-Dimethoxy-3-phenyl-3*H*-chromene[3,4-c]quinoline (**7b**). Yellow solid, η 63%, m.p. 127–128 °C. ^1^H-NMR (500.13 MHz, CDCl_3_): δ 3.95 (s, 3H, 11-OC*H*_3_); 4.07 (s, 3H, 10-OC*H*_3_); 6.34 (s, 1H, H-6); 6.91 (s, 1H, H-12); 7.04 (dd, 1H, H-4, *J* 1.0 and 8.1 Hz); 7.16 (dt, 1H, H-2, *J* 1.0 and 7.6 Hz); 7.30 (s, 1H, H-7); 7.35 (ddd, 1H, H-3, *J* 1.6; 7.6 and 8.1 Hz); 7.40–7.52 (m, 6H, H-9,2′,3′,4′,5′,6′); 8.45 (dd, 1H, H-1, *J* 1.6 and 7.6 Hz) ppm. ^13^C-NMR (125.77 MHz, CDCl_3_): δ 56.0 (11-O*C*H_3_); 56.2 (10-O*C*H_3_); 80.4 (C-6); 105.0 (C-12a); 105.3 (C-12); 107.9 (C-9); 117.6 (C-4); 122.5 (C-2); 123.1 (C-12c); 125.0 (C-1); 126.7 (C-6a); 128.3 (C-2′,6′); 128.8 (C-3′,5′); 128.9 (C-4′); 131.2 (C-3); 131.4 (C-7); 139.0 (C-1′); 145.1 (C-8a); 146.8 (C-12b); 149.7 (C-11); 152.7 (C-10); 156.1 (C-4a) ppm. ESI^+^-MS *m/z* (%): 370.1 [M *+* H]^+^ (100). EI-HRMS: calcd. for (C_24_H_19_NO_3_) 369.1365; found 369.1368.

10-Nitro-3-phenyl-3*H*-chromeno[3,4-c]quinoline (**7c**). Orange oil, η 25%. ^1^H-NMR (500,13 MHz, CDCl_3_): δ 6.38 (s, 1H, H-6); 7.09 (d, 1H, H-4, *J* 8.0 Hz); 7.20–7.26 (m, 1H, H-2); 7.44–7.49 (m, 6H, H-3, 2′,3′,4′,5′,6′), 7.60 (s, 1H, H-7); 8.26 (d, 1H, H-9, *J* 9.1 Hz); 8.45 (dd, 1H, H-10, *J* 2.4 and 9.1 Hz); 8.55 (d, 1H, H-1, *J* 7.5 Hz); 8.62 (d, 1H, H-12, *J* 2.4 Hz) ppm. ^13^C-NMR (500,13 MHz, CDCl_3_): δ 80.2 (C-6); 118.0 (C-4); 122.2 (C-12a); 122.6 (C-2); 123.1 (C-10); 123.5 (C-6a); 124.6 (C-12); 126.4 (C-1); 128.2 (C-2′,6′); 128.6 (C-12c); 129.1 (C-3′,5′); 129.4 (C-4′); 130.8 (C-9); 133.5 (C-3); 134.5 (C-7); 137.8 (C-1′); 145.1 (C-11); 150.2 (C-8a); 152.4 (C-12b); 157.2 (C-4a) ppm. ESI^+^-MS *m/z* (%):355.2 [M *+* H]^+^ (100).

3-(4-Methoxyphenyl)-3*H*-chromeno[3,4-c]quinoline (**7d**). Yellow oil, η 50%. ^1^H-NMR (500,13 MHz, CDCl_3_): δ 3.84 (s, 3H, 4′-OC*H*_3_); 6.32 (s, 1H, H-6); 6.96 (d, 2H, H-3′,5′, J 8.8 Hz); 7.03 (d, 1H, H-4, J 9.2 Hz); 7.17 (t, 1H, H-2, J 7.5 Hz); 7.33–7.50 (m, 5H, H-3, 7, 11, 2′,6′); 7.65 (d, 1H, H-12, J 7.8 Hz); 7.69 (t, 1H, H-10, J 7.7 Hz); 8.14 (d, 1H, H-9, J 7.8 Hz); 8.52 (d, 1H, H-1, J 9.2 Hz) ppm. ^13^C-NMR (500,13 MHz, CDCl_3_): δ 55.4 (4′-O*C*H_3_); 80.0 (C-6); 114.2 (C-3′,5′); 117.7 (C-4); 122.5 (C-2); 123.5 (C-12c); 125.5 (C-1); 126.2 (C-11); 127.6 (C-12); 127,7 (C-12a); 128.8 (C-12); 129.3 (C-10); 129.7 (C-2′,6′); 129.7 (C-1′); 130.8 (C-1′); 132.0 (C-3); 132.9 (C-7); 148.1 (C-8a); 149.0 (C-12b); 156.5 (C-4a); 160.0 (C-4′) ppm. ESI^+^-MS *m/z* (%): 340.2 [M *+* H]^+^ (100).

10,11-Dimethoxy-3-(4-methoxyphenyl)-3*H*-chromene[3,4-c]quinoline (**7e**). Yellow solid, η 75%, m.p. 107–108 °C. ^1^H-NMR (300.13 MHz, acetone-*d*_6_): δ 3.82 (s, 3H, 4′-OC*H*_3_); 3.93 (s, 3H, 11-OC*H*_3_); 4.02 (s, 3H, 10-OC*H*_3_); 6.42 (s, 1H, H-6); 6.94–7.02 (m, 3H, H-4,3′,5′); 7.14 (dt, 1H, H-2, *J* 1.1 and 7.7 Hz); 7.20 (s, 1H, H-12); 7.32–7.43 (m, 3H, H-3,2′,6′); 7.44 (s, 1H, H-9); 7.53 (s, 1H, H-7); 8.44 (dd, 1H, H-1, *J* 1.6 and 7.7 Hz) ppm. ^13^C-NMR (75.47 MHz, acetone-*d*_6_): δ 55.5 (4′-O*C*H_3_); 56.1 (10- and 11-O*C*H_3_); 80.2 (C-6); 106.4 (C-9); 108.5 (C-12); 114.7 (C-3′,5′); 118.3 (C-4); 122.8 (C-2); 124.1 (C-12a); 124.5 (C-12c); 125.6 (C-1); 127.3 (C-6a); 130.3 (C- 2′,6′); 131.9 (C-3,7); 132.5 (C-1′); 145.9 (C-8a); 146.8 (C-12b); 151.0 (C-11); 154.0 (C-10); 156.9 (C-4a); 160.8 (C-4′) ppm. ESI^+^-MS *m/z* (%): 400.1 [M *+* H]^+^ (100). EI-HRMS: calcd. for (C_25_H_21_NO_4_) 399.1471; found 399.1475.

10,11-Dimethoxy-3-(4-nitrophenyl)-3*H*-chromene[3,4-c]quinoline (**7f**). Yellow solid, η 73%, m.p. 149–150 °C. ^1^H-NMR (300.13 MHz, CDCl_3_): δ 3.97 (s, 3H, 10-OC*H*_3_); 4.07 (s, 3H, 11-OC*H*_3_); 6.44 (s, 1H, H-6); 6.93 (s, 1H, H-9); 7.04 (dd, 1H, H-4, *J* 1.0 and 7.8 Hz); 7.18 (dt, 1H, H-2, *J* 1.0 and 7.8 Hz); 7.32 (s, 1H, H-7); 7.37 (dt, 1H, H-3, *J* 1.6 and 7.8 Hz); 7.50 (s, 1H, H-12); 7.65 (d, 2H, H-2′,6′, *J* 8.8 Hz); 8.28 (d, 2H, H-3′,5′, *J* 8.8 Hz); 8.44 (dd, 1H, H-1, *J* 1.6 and 7.8 Hz) ppm. ^13^C-NMR (75.47 MHz, CDCl_3_): δ 56.1 (10- O*C*H_3_); 56.3 (11-O*C*H_3_); 78.9 (C-6); 105.2 (C-9); 107.9 (C-12); 117.6 (C-4); 123.0 (C-12a); 123.0 (C-2); 123.3 (C-12c); 123.9 (C-3′,5′); 125.0 (C-6a); 125.1 (C-1); 128.9 (C-2′,6′); 131.0 (C-7); 131.6 (C-3); 145.4 (C-8a); 146.1 (C-1′); 146.2 (C-12b); 148.1 (C-4′); 150.0 (C- 10); 153.0 (C-11); 155.3 (C-4a) ppm. ESI^+^-MS *m/z* (%):415.1 [M *+* H]^+^ (100). EI-HRMS: calcd. for (C_24_H_18_N_2_O_5_) 414.1216; found 414.1219.

### 3.5. General Procedures for the Synthesis of the (Z)-and (E)-2-aryl-4-chloro-3-styryl-2H-chromenes ***10a**–**i/11a**–**i***

A mixture of appropriate phosphonium halide (**8a**–**c**, 77 mg, 0.21 mmol) in freshly dry THF (10 mL) and NaH (13 mg, 0.5 mmol) was stirred under reflux until formation of the corresponding ylide, then, the 4-chloro-2-phenyl-2*H*-chromene-3-carbaldehydes **2a**–**c** (0.07 mmol) was added and the reaction mixture continued in reflux (or room temperature). At the end, the reaction mixture was poured into a mixture of ice (10 g) and water (20 mL) and acidified at pH ~3 with a solution HCl (10%). Then, extracted with ethyl acetate (3 × 20 mL), the organic layer was dried over anhydrous sodium sulphate, the solvent was evaporated to dryness and the solid residue was purified by thin layer chromatography using as eluent a mixtures dichloromethane/hexane.

(Z)-4-Chloro-2-phenyl-3-styryl-2*H*-chromene (**10a**). White solid, η 32%, m. p. 112–113 °C. ^1^H-NMR (300.13 MHz; CDCl_3_): δ 5.89 (s, 1H, H-2); 6.37 (d, 1H, H-α, *J* 12.3 Hz); 6.64 (d, 1H, H-β, *J* 12.3 Hz); 6.76 (dd, 1H, H-8, *J* 1.3 and 8.0 Hz); 6.95 (ddd, 1H, H-6, *J* 1.3, 7.1 and 8.0 Hz); 7.14–7.22 (m, 6H, H-2′,3′,4′,5′,6′,7); 7.31–7.43 (m, 5H, H-2″,3″, 4″,5″,6″); 7.55 (dd, 1H, H-5, *J* 1.3 and 8.0 Hz) ppm. ^13^C-NMR (75.47 MHz; CDCl_3_): δ 78.2 (C-2); 116.4 (C-8); 121.3 (C-4a); 121.4 (C-6); 124.5 (C-β); 124.6 (C-5); 126.8 (C-4); 127.2 (C-2′,6′); 128.1 (C-4′); 128.3 (C-3′,5′); 128.4 (C-4″); 128.5 (C-2″,6″); 128.6 (C-3″,5″); 128.7 (C-1′); 129.1 (C-3); 130.4 (C-7); 133.3 (C-α); 136.7 (C-1″); 152.7 (C-8a) ppm. ESI^+^-MS *m/z* (%): 367.1 Cl^35^ [M *+* Na]^+^ (45), 369.1 Cl^37^ [M *+* Na]^+^ (15). EI-HRMS *m/z* calcd for (C_23_H_17_^35^ClO) 344.0968, found 344.0964. Calcd for (C_23_H_17_^37^ClO) 346.0938, found 346.0933.

(E)-4-Chloro-2-phenyl-3-styryl-2*H*-chromene (**11a**). White solid, η 25%, m. p. 121–123 °C. ^1^H-NMR (300.13 MHz; CDCl_3_): δ 6.33 (s, 1H, H-2); 6.49 (d, 1H, H-β, *J* 16.5 Hz); 6.79 (dd, 1H, H-8, *J* 1.3 and 8.0 Hz); 6.94 (ddd, 1H, H-6, *J* 1.3, 7.1 and 8.0 Hz); 7.13 (ddd, 1H, H-7, *J* 1.3, 7.1 and 8.0 Hz); 7.25–7.34 (m, 6H, H-2″,3″,4″,5″,6″,4′); 7.39–7.45 (m, 4H, H-2′,3′,5′,6′); 7.56 (d, 1H, H-α, *J* 16.5 Hz); 7.57 (dd, 1H, H-5, *J* 1.3 and 8.0 Hz) ppm. ^13^C-NMR (75.47 MHz; CDCl_3_): δ 77.4 (C-2); 116.7 (C-8); 121.7 (C-6); 122.2 (C-4a); 123.2 (C-α); 125.0 (C-5); 126.8 (C-2″,6″); 127.2 (C-4); 127.7 (C-2′,6′); 128.3 (C-4′); 128.5 (C-3″,5″); 128.7 (C-3′,5′); 130.4 (C-7); 131.8 (C-β); 136.7 (C-3); 137.7 (C-1′); 152.2 (C-8a) ppm.

(Z)-4-Chloro-3-(4-ethoxyvinyl)-2-phenyl-2*H*-chromene (**10b**). White solid, η 45%, m. p. 100–102 °C. ^1^H-NMR (300.13 MHz; CDCl_3_): δ 1.42 (t, 3H, 4″-OCH_2_C*H*_3_, *J* 7.0 Hz); 4.05 (qd, 2H, 4′-OC*H*_2_CH_3_, *J* 1.7 and 7.0 Hz); 5.95 (s, 1H, H-2); 6.24 (d, 1H, H-α, *J* 12.2 Hz); 6.58 (d, 1H, H-β, *J* 12.2 Hz); 6.77 (dd, 1H, H-8, *J* 1.1 and 7.8 Hz); 6.87 (d, 2H, H-3″,5″, *J* 9.0 Hz); 6.95 (dt, 1H, H-6, *J* 1.1 and 7.6 Hz); 7.15 (dt, 1H, H-7, *J* 1.1 and 7.6 Hz); 7.19–7.23 (m, 5H, H-2′,3′,4′,5′,6′); 7.37 (d, 2H, H-2″,6″, *J* 9.0 Hz); 7.54 (dd, 1H, H-5, *J* 1.1 and 7.8 Hz) ppm. ^13^C-NMR (75.47 MHz; CDCl_3_): δ 14.8 (4″-OCH_2_*C*H_3_); 63.4 (4″-O*C*H_2_CH_3_); 78.2 (C-2); 114.4 (C-3″,5″); 116.3 (C-8); 121.4 (C-6); 121.5 (C-4a); 122.7 (C-α); 124.5 (C-5); 126.3 (C-4); 127.2 (C-2′,6′); 128.2 (C-3′,5′); 128.4 (C-4′); 129.1 (C-1′); 129.5 (C-3); 130.1 (C-2″,6″); 130.2 (C-7); 132.9 (C-β); 138.9 (C-1″); 152.6 (C-8a); 158.8 (C-4″) ppm. ESI^+^-MS *m/z* (%): 411.1 Cl^35^ [M *+* Na]^+^ (100); 413.1 Cl^37^ [M *+* Na]^+^ (3). EI-HRMS *m/z* calcd for (C_25_H_21_^35^ClO_2_) 388.1230, found 388.1226. Calcd for (C_25_H_21_^37^ClO_2_) 390.1201, found 390.1216.

(E)-4-Chloro-3-(4-ethoxyvinyl)-2-phenyl-2*H*-chromene (**11b**). Yellow solid, η 25%, m. p. 166–167 °C. ^1^H-NMR (300.13 MHz; CDCl_3_): δ 1.41 (t, 3H, 4″-OCH_2_CH_3_, *J* 7.0 Hz); 4.02 (q, 2H, 4′-OCH_2_CH_3_, *J* 7.0 Hz); 6.31 (s, 1H, H-2); 6.44 (d, 1H, H-β, *J* 16.5 Hz); 6.78 (d largo, 1H, H-8, *J* 8.0 Hz); 6.85 (d, 2H, H-3″,5″, *J* 9.1 Hz); 6.94 (dt, 1H, H-6, *J* 1.4 and 7.7 Hz); 7.11 (dt, 1H, H-7, *J* 1.4 and 7.7 Hz); 7.26–7.29 (m, 3H, H-3′,4′,5′); 7.37 (d, 2H, H-2″,6″, *J* 9.1 Hz); 7.39–7.42 (m, 2H, H-2′,6′); 7.42 (d, 1H, H-α, *J* 16.5 Hz); 7.55 (dd, 1H, H-5, *J* 1.4 and 8.0 Hz) ppm. ^13^C-NMR (75.47 MHz; CDCl_3_): δ 14.8 (4″-OCH_2_*C*H_3_); 63.5 (4″-O*C*H_2_CH_3_); 77.1 (C-2); 114.7 (C-3″,5″); 116.6 (C-8); 121.0 (C-α); 121.6 (C-6); 122.4 (C-4a); 124.9 (C-5); 125.8 (C-4); 127.7 (C-3′,5′); 127.8 (C-3); 128.1 (C-2″,6″); 128.4 (C-1″); 128.5 (C-2′,6′); 128.6 (C-4′); 129.4 (C-1′); 130.0 (C-7); 131.5 (C-β); 137.8 (C-3); 152.1 (C-8a); 159.3 (C-4″) ppm.

(Z)-4-Chloro-2-phenyl-3-(4-nitrovinyl)-2*H*-chromene (**10c**). Yellow solid, η 41%, m. p. 159–161 °C. ^1^H-NMR (300.13 MHz; CDCl_3_): δ 5.78 (s, 1H, H-2); 6.53 (d, 1H, H-α, *J* 12.2 Hz); 6.66 (d, 1H, H-β, *J* 12.2 Hz); 6.79 (dd, 1H, H-8, *J* 1.2 and 7.9 Hz); 6.97 (dt, 1H, H-6, *J* 1.2 and 7.6 Hz); 7.16–7.25 (m, 6H, H-7, 2′,3′,4′,5′,6′); 7.53 (d, 2H, H-2″,6″, *J* 8.8 Hz); 7.55 (d, 1H, H-5, *J* 7.9 Hz); 8.17 (d, 2H, H-3″,5″, *J* 8.8 Hz) ppm. ^13^C-NMR (75.47 MHz; CDCl_3_): δ 78.5 (C-2); 116.5 (C-8); 121.7 (C-6); 121.9 (C-4a); 123.8 (C-3″,5″); 125.5 (C-5); 127.2 (C-2′,6′); 127.6 (C-4); 128.3 (C-β); 128.5 (C-3′,5′); 128.7 (C-4′); 129.0 (C-1′); 129.2 (C-2″,6″); 130.9 (C-α); 131.0 (C-7); 138.2 (C-3); 143.3 (C-1″); 147.0 (C-4″); 152.4 (C-8a) ppm. ESI^+^-MS *m/z* (%): 413.3 Cl^35^ [M *+* Na]^+^ (100), 415.3 Cl^37^ [M *+* Na]^+^ (30). Anal. Calcd for C_23_H_16_ClNO_3_: C, 70,86%; H, 4,14%; N, 3,59%. Found: C, 71,19%; H, 4,16%; N, 3,53%.

(E)-4-Chloro-2-phenyl-3-(4-nitrovinyl)-2*H*-chromene (**11c**). Yellow solid, η 33%, m. p. 194–196 °C. ^1^H-NMR (300.13 MHz; CDCl_3_): δ 6.32 (s, 1H, H-2); 6.50 (d, 1H, H-β, *J* 16.5 Hz); 6.80 (dd, 1H, H-8, *J* 1.3 and 8.0 Hz); 6.97 (ddd, 1H, H-6, *J* 1.3, 7.2 and 8.0 Hz); 7.18 (ddd, 1H, H-7, *J* 1.3, 7.2 and 8.0 Hz); 7.28–7.33 (m, 3H, H-3′,4′,5′); 7.39-7.42 (m, 2H, H-2′,6′); 7.56 (d, 2H, H-2″,6″, *J* 9.0 Hz); 7.60 (dd, 1H, H-5, *J* 1.3 and 8.0 Hz); 7.71 (d, 1H, H-α, *J* 16.5 Hz); 8.18 (d, 2H, H-3″,5″, *J* 9.0 Hz) ppm. ^13^C-NMR (75.47 MHz; CDCl_3_): δ 77.2 (C-2); 116.9 (C-8); 121.7 (C-4a); 121.9 (C-6); 124.1 (C-3″,5″); 125.5 (C-5); 126.4 (C-4); 127.2 (C-2″,6″); 127.3 (C-α); 127.6 (C-2′,6′); 128.7 (C-3′,5′); 128.8 (C-4′); 129.0 (C-β); 129.2 (C-1′); 131.0 (C-7); 137.2 (C-3); 143.1 (C-1″); 146.8 (C-4″); 152.5 (C-8a) ppm.

(Z)-4-Chloro-2-(4-methoxyphenyl)-3-vinyl-2*H*-chromene (**10d**). Yellow solid, η 50%, m. p. 78–80 °C. ^1^H-NMR (300.13 MHz; CDCl_3_): δ 3.73 (s, 3H, 4′-OCH_3_); 5.83 (s, 1H, H-2); 6.35 (d, 1H, H-α, *J* 12.2 Hz); 6.63 (d, 1H, H-β, *J* 12.2 Hz); 6.72 (dlargo, 1H, H-8, *J* 8.0 Hz); 6.73 (d, 2H, H-3′,5′, *J* 8.9 Hz); 6.95 (dt, 1H, H-6, *J* 1.2 and 7.6 Hz); 7.06 (d, 2H, H-2′,6′, *J* 8.9 Hz); 7.11 (dt, 1H, H-7, *J* 1.2 and 7.6 Hz); 7.29–7.42 (m, 5H, H-2″,3″,4″,5″,6″); 7.55 (dd, 1H, H-5, *J* 1.2 and 8.0 Hz) ppm. ^13^C-NMR (75.47 MHz; CDCl_3_): δ 55.1 (4′-OCH_3_); 77.9 (C-2); 113.6 (C-3′,5′); 116.4 (C-8); 121.3 (C-6); 121.4 (C-4a); 124.4 (C-α); 124.5 (C-5); 126.8 (C-4); 128.1 (C-4″); 128.5 (C-3″,5″); 128.6 (C-2″,6″); 128.7 (C-2′,6′); 129.3 (C-3); 130.3 (C-7); 130.8 (C-1′); 133.3 (C-β); 136.7 (C-1″); 152.5 (C-8a); 159.7 (C-4′) ppm. ESI^+^-MS *m/z* (%): 413.1 Cl^35^ [M *+* K]^+^ (10). EI-HRMS *m/z* calcd for (C_24_H_19_^35^ClO_2_) 374.1074; found 374.1082. Calcd for (C_24_H_19_^37^ClO_2_) 376.1044; found 376.1050.

(E)-4-Chloro-2-(4-methoxyphenyl)-3-vinyl-2*H*-chromene (**11d**). White solid, η 28%, m. p. 187–189 °C. ^1^H-NMR (300.13 MHz; CDCl_3_): δ 3.73 (s, 3H, 4′-OC*H*_3_); 6.28 (s, 1H, H-2); 6.47 (d, 1H, H-β, *J* 16.5 Hz); 6.77 (dd, 1H, H-8, *J* 1.4 and 8.3 Hz); 6.80 (d, 2H, H-3′,5′, *J* 8.7 Hz); 6.95 (dt, 1H, H-6, *J* 1.4 and 7.6 Hz); 7.13 (dt, 1H, H-7, *J* 1.4 and 7.6 Hz); 7.24–7.29 (m, 1H, H-4″); 7.31-7.35 (m, 4H, H-2′,6′,3″,5″); 7.42–7.45 (m, 2H, H-2″,6″); 7.44 (d, 2H, H-2′,6′, *J* 8.7 Hz); 7.53 (d, 1H, H-α, *J* 16.5 Hz); 7.58 (dd, 1H, H-5, *J* 1.4 and 8.3 Hz) ppm. ^13^C-NMR (75.47 MHz; CDCl_3_): δ 55.1 (4′-O*C*H_3_); 76.9 (C-2); 113.9 (C-3′,5′); 116.7 (C-8); 121.6 (C-6); 122.2 (C-4a); 123.0 (C-α); 125.0 (C-5); 126.8 (C-2″,6″); 127.4 (C-4); 128.3 (C-4″); 128.5 (C-3); 128.7 (C-2′,6′); 129.1 (C-3″,5″); 129.8 (C-1′); 130.3 (C-7); 131.8 (C-β); 136.7 (C-1″); 152.2 (C-8a); 159.8 (C-4′) ppm.

(*Z*)-4-Chloro-3-(4-ethoxyvinyl)-2-(4-methoxyphenyl)-2*H*-chromene (**10e**). Yellow oil, η 30%. ^1^H-NMR (300.13 MHz; CDCl_3_): δ 1.43 (t, 3H, 4″-OCH_2_C*H*_3_, *J* 7.0 Hz); 3.73 (s, 1H, 4′-OC*H*_3_); 4.05 (q, 2H, 4″-OC*H*_2_CH_3_, *J* 7.0 Hz); 5.89 (s, 1H, H-2); 6.22 (d, 1H, H-α, *J* 12.1 Hz); 6.56 (d, 1H, H-β, *J* 12.1 Hz); 6.72–6.75 (m, 3H, H-8,3′,5′); 6.87 (d, 2H, H-3″,5″, *J* 8.9 Hz); 6.95 (dt, 1H, H-6, *J* 1.4 and 7.7 Hz); 7.09 (d, 2H, H-2′,6′, *J* 8.9 Hz); 7.14 (dt, 1H, H-7, *J* 1.4 and 7.7 Hz); 7.36 (d, 2H, H-2″,6″, *J* 8.9 Hz); 7.55 (dd,1H, H-5, *J* 1.4 and 8.0 Hz) ppm. ^13^C-NMR (75.47 MHz; CDCl_3_): δ 14.8 (4″-OCH_2_*C*H_3_); 55.1 (4′-O*C*H_3_); 63.4 (4″-O*C*H_2_CH_3_); 77.9 (C-2); 113.6 (C-3′,5′); 114.6 (C-3″,5″); 116.4 (C-8); 121.3 (C-6); 121.4 (C-4a); 122.6 (C-α); 124.4 (C-5); 126.2 (C-4); 128.7 (C-2′,6′); 129.1 (C-1″); 129.7 (C-3); 130.0 (C-2″,6″); 130.2 (C-7); 130.9 (C-1′); 132.9 (C-β); 152.5 (C-8a); 158.8 (C-4″); 159.6 (C-4′) ppm. ESI^+^-MS *m/z* (%): 441.2 Cl^35^ [M *+* Na]^+^ (10); 443.2 Cl^37^ [M *+* Na]^+^ (10). Anal. Calcd for C_26_H_23_ClO_3_: C, 74.55%; H, 5.53%. Found C, 74.73%; H, 5.55%.

(*E*)-4-Chloro-3-(4-ethoxyvinyl)-2-(4-methoxyphenyl)-2*H*-chromene (**11e**). Yellow solid, η 20%, m. p. 140–142 °C. ^1^H-NMR (300.13 MHz; CDCl_3_): δ 1.41 (t, 3H, 4″-OCH_2_C*H*_3_, *J* 7.0 Hz); 3.73 (s, 1H, 4′-OC*H*_3_); 4.03 (q, 2H, 4″-OC*H*_2_CH_3_, *J* 7.0 Hz); 6.26 (s, 1H, H-2); 6.42 (d, 1H, H-β, *J* 16.5 Hz); 6.76 (dd, 1H, H-8, *J* 1.3 and 8.0 Hz); 6.79 (d, 2H, H-3′,5′, *J* 8.8 Hz); 6.84 (d, 2H, H-3″,5″, *J* 8.8 Hz); 6.93 (dt, 1H, H-6, *J* 1.3 and 7.6 Hz); 7.11 (dt, 1H, H-7, *J* 1.3 and 7.6 Hz); 7.32 (d, 2H, H-2′,6′, *J* 8.8 Hz); 7.37 (d, 2H, H-2″,6″, *J* 8.8 Hz); 7.42 (d, 1H, H-α, *J* 16.5 Hz); 7.56 (dd, 1H, H-5, *J* 1.3 and 8.0 Hz) ppm. ^13^C-NMR (75.47 MHz; CDCl_3_): δ 14.7 (4″-OCH_2_*C*H_3_); 55.1 (4′-O*C*H_3_); 63.5 (4″-O*C*H_2_CH_3_); 76.9 (C-2); 113.8 (C-3′,5′); 114.6 (C-3″,5″); 116.6 (C-8); 120.9 (C-α); 121.5 (C-6); 122.4 (C-4a); 124.8 (C-5); 127.7 (C-4); 128.1 (C-2″,6″); 128.7 (C-3); 129.1 (C-2′,6′); 129.4 (C-1″); 129.9 (C-1′); 130.0 (C-7); 131.4 (C-β); 152.0 (C-8a); 159.2 (C-4″); 159.7 (C-4′) ppm.

(*Z*)-4-Choro-2-(4-methoxyphenyl)-3-(4-nitrovinyl)-2*H*-chromene (**10f**). Yellow oil, η 53%. ^1^H-NMR (300.13 MHz; CDCl_3_): δ 3.74 (s, 3H, 4-OCH_3_); 5.72 (s, 1H, H-2); 6.51 (d, 1H, H-α, *J* 12.3 Hz); 6.64 (d, 1H, H-β, *J* 12.3 Hz); 6.78 (dlargo, 1H, H-8, *J* 8.0 Hz); 6.82 (d, 2H, H-3′,5′, *J* 8.7 Hz); 6.97 (dt, 1H, H-6, *J* 1.3 and 7.6 Hz); 7.18 (td, 1H, H-7, *J* 1.3 and 7.6 Hz); 7.31 (d, 2H, H-2′,6′, *J* 8.7 Hz); 7.55 (d, 2H, H-2″,6″, *J* 8.7 Hz); 7.61 (d, 1H, H-5, *J* 8.0 Hz); 8.17 (d, 2H, H-3″,5″, *J* 8.8 Hz) ppm. ^13^C-NMR (75.47 MHz; CDCl_3_): δ 55.2 (4′-OCH_3_); 78.2 (C-2); 114.0 (C-3′,5′); 116.9 (C-8); 121.6 (C-4a); 121.8 (C-6); 124.1 (C-3″,5″); 125.4 (C-5); 126.5 (C-4); 127.1 (C-2″,6″); 127.2 (C-4′); 128.2 (C-α); 129.0 (C-2′,6′); 129.2 (C-1′); 129.3 (C-3); 131.0 (C-β); 131.2 (C-7); 143.2 (C-1″); 147.0 (C-4″); 152.4 (C-8a); 160.0 (C-4′) ppm. ESI^+^-MS *m/z* (%): 442.1 Cl^35^ [M *+* Na]^+^ (20); 444.1 Cl^37^ [M *+* Na]^+^ (10). EI-HRMS *m/z* calcd for (C_24_H_18_N^35^ClO_4_) 419.0924, found 419.0932. Calcd for (C_24_H_18_N^37^ClO_4_) 421.0895, found 421.0895.

(*E*)-4-Chloro-2-(4-methoxyphenyl)-3-(4-nitrovinyl)-2*H*-chromene (**11f**). Yellow solid, η 35%, m. p. 170–172 °C. ^1^H-NMR (300.13 MHz; CDCl_3_): δ 3.75 (s, 3H, 4′-OC*H*_3_); 6.27 (s, 1H, H-2); 6.47 (d, 1H, H-β, *J* 16.6 Hz); 6.78 (dd, 1H, H-8, *J* 1.3 and 8.0 Hz); 6.82 (d, 2H, H-3′,5′, *J* 8.8 Hz); 6.97 (dt, 1H, H-6, *J* 1.3 and 7.6 Hz); 7.17 (dt, 1H, H-7, *J* 1.3 and 7.6 Hz); 7.31 (d, 2H, H-2′,6′, *J* 8.8 Hz); 7.55 (d, 2H, H-2″,6″, *J* 8.8 Hz); 7.61 (dd, 1H, H-5, *J* 1.3 and 8.0 Hz); 7.68 (d, 1H, H-α, *J* 16.6 Hz); 8.17 (d, 2H, H-3″,5″, *J* 8.8 Hz) ppm. ^13^C-NMR (75.47 MHz; CDCl_3_): δ 55.2 (4′-O*C*H_3_); 76.8 (C-2); 114.0 (C-3′,5′); 116.9 (C-8); 121.6 (C-4a); 121.8 (C-6); 124.1 (C-3″,5″); 125.4 (C-5); 126.4 (C-4); 127.1 (C-2′,6′); 127.2 (C-α); 128.7 (C-3); 128.9 (C-β); 129.0 (C-2″,6″); 129.2 (C-1′); 131.2 (C-7); 143.4 (C-1″); 147.0 (C-4″); 152.4 (C-8a); 160.0 (C-4′) ppm.

(*Z*)-4-Chloro-2-(4-nitrophenyl)-3-vinyl-2*H*-chromene (**10g**). Red oil, η 34%. ^1^H-NMR (300.13 MHz; CDCl_3_): δ 5.30 (s, 1H, H-2); 6.48 (d, 1H, H-α, *J* 12.2 Hz); 6.72 (d, 1H, H-β, *J* 12.2 Hz); 6.80 (dd, 1H, H-8, *J* 1.3 and 8.0 Hz); 6.99 (dt, 1H, H-6, *J* 1.3 and 7.6 Hz); 7.20 (dt, 1H, H-7, *J* 1.3 and 7.6 Hz); 7.30 (d, 2H, H-2′,6′, *J* 9.0 Hz); 7.33-7.39 (m, 5H, H-2″,3″,4″,5″,6″); 7.56 (dd, 1H, H-5, *J* 1.3 and 8.0 Hz); 8.05 (d, 2H, H-3′,5′, *J* 9.0 Hz) ppm. ^13^C-NMR (75.47 MHz; CDCl_3_): δ 77.3 (C-2); 116.2 (C-8); 121.0 (C-4a); 121.9 (C-6); 123.3 (C-3′,5′); 124.0 (C-α); 124.8 (C-5); 126.7 (C-3); 127.7 (C-4); 127.8 (C-2′,6′); 128.3 (C-4″); 128.4 (C-3″,5″); 128.5 (C-2″,6″); 130.7 (C-7); 133.7 (C-β); 136.3 (C-1″); 145.8 (C-4′); 147.5 (C-1′); 152.1 (C-8a) ppm. ESI^+^-MS *m/z* (%): 412.1 Cl^35^ [M *+* Na]^+^ (15); 414.3 Cl^37^ [M *+* Na]^+^ (10). EI-HRMS *m/z* calcd for (C_23_H_16_N^35^ClO_3_) 389.0819, found 389.0817. Calcd for (C_23_H_16_N^37^ClO_3_) 391.0789; found 391.0804.

(*E*)-4-Chloro-2-(4-nitrophenyl)-3-vinyl-2*H*-chromene (**11g**). Orange oil, η 15%. ^1^H-NMR (300.13 MHz; CDCl_3_): δ 6.38 (s, 1H, H-2); 6.48 (d, 1H, H-β, *J* 16.5 Hz); 6.84 (dd, 1H, H-8, *J* 1.2 and 8.1 Hz); 6.99 (ddd, 1H, H-6, *J* 1.2, 7.0 and 8.1 Hz); 7.17 (ddd, 1H, H-7, *J* 1.2, 7.0 and 8.1 Hz); 7.28–7.37 (m, 5H, H-2″,3″,4″,5″,6″); 7.57 (dlargo, 1H, H-5, *J* 8.1 Hz); 7.60 (d, 2H, H-2′,6′, *J* 9.0 Hz); 7.61 (d, 1H, H-α, *J* 16.5 Hz); 8.14 (d, 2H, H-3′,5′, *J* 9.0 Hz) ppm. ^13^C-NMR (75,47 MHz; CDCl_3_): δ 75.6 (C-2); 116.7 (C-8); 122.0 (C-4a); 122.3 (C-6); 123.0 (C-α); 123.8 (C-2′,6′); 125.4 (C-5); 126.3 (C-4); 126.9 (C-2″6″); 127.7 (C-1″); 128.7 (C-3′,5′); 128.8 (C-4″); 128.9 (C-3″,5″); 130.8 (C-7); 132.0 (C-β); 136.3 (C-3); 144.9 (C-1′); 148.0 (C-4′); 151.8 (C-8a) ppm.

(*Z*)-4-Chloro-3-(4-ethoxyvinyl)-2-(4-nitrophenyl)-2*H*-chromene (**10h**). Yellow oil, η 45%. ^1^H-NMR (300.13 MHz; CDCl_3_): δ 1.43 (t, 3H, 4″-OCH_2_C*H*_3_, *J* 7.0 Hz); 4.06 (q, 2H, 4″-OC*H*_2_CH_3_, *J* 7.0 Hz); 6.03 (s, 1H, H-2); 6.34 (d, 1H, H-α, *J* 12.1 Hz); 6.65 (d, 1H, H-β, *J* 12.1 Hz); 6.82 (dd, 1H, H-8, *J* 1.3 and 8.0 Hz); 6.87 (d, 2H, H-3″,5″, *J* 9.0 Hz); 6.99 (dt, 1H, H-6, *J* 1.3 and 7.6 Hz); 7.20 (dt, 1H, H-7, *J* 1.3 and 7.6 Hz); 7.34 (d, 4H, H-2′,6′,2″,6″, *J* 9.0 Hz); 7.55 (dd, 1H, H-5, *J* 1.3 and 8.0 Hz); 8.05 (d, 2H, H-3′,5′, *J* 9.0 Hz) ppm ^13^C-NMR (75.47 MHz; CDCl_3_): δ 14.8 (4″-OCH_2_*C*H_3_); 63.5 (4″-O*C*H_2_CH_3_), 76.7 (C-2); 114.5 (C-3″,5″); 116.3 (C-8); 121.3 (C-4a); 122.0 (C-6); 122.3 (C-α); 123.5 (C-3′,5′); 124.8 (C-5); 127.5 (C-3); 127.9 (C-2″,6″); 128.3 (C-4); 128.7 (C-1″); 130.1 (C-2′,6′); 130.7 (C-7); 133.6 (C-β); 146.2 (C-4′); 147.4 (C-1′); 152.2 (C-8a); 159.1 (C-4″) ppm. ESI^+^-MS *m/z* (%): 456.1 ^35^Cl [M *+* Na]^+^ (45); 458.1 ^37^Cl [M *+* Na]^+^ (15). EI^+^-HRMS *m/z* calcd for (C_25_H_20_NO_4_^35^Cl) 433.1081; found 433.1084. Calcd for (C_25_H_20_NO_2_^37^Cl) 435.1051; found 435.1060.

(*E*)-4-Chloro-3-(4-ethoxyvinyl)-2-(4-nitrophenyl)-2*H*-chromene (**11h**). Yellow oil, η 24%. ^1^H-NMR (300.13 MHz; CDCl_3_): δ 1.43 (t, 3H, 4″-OCH_2_C*H*_3_, *J* 7.0 Hz); 4.06 (q, 2H, 4″-OC*H*_2_CH_3_, *J* 7.0 Hz); 6.36 (s, 1H, H-2); 6.42 (d, 1H, H-β, *J* 16.5 Hz); 6.83 (dd, 1H, H-8, *J* 1.4 and 8.0 Hz); 6.87 (d, 2H, H-3″,5″, *J* 9.0 Hz); 6.97 (dt, 1H, H-6, *J* 1.4 and 7.8 Hz); 7.15 (dt, 1H, H-7, *J* 1.4 and 7.8 Hz); 7.39 (d, 2H, H-2″,6″, *J* 9.0 Hz); 7.46 (d, 1H, H-α, *J* 16.5 Hz); 7.56 (d, 1H, H-5, *J* 1.4 and 8.0 Hz); 7.60 (d, 2H, H-2′,6′, *J* 9.0 Hz); 8.13 (d, 2H, H-3′,5′, *J* 9.0 Hz) ppm. ^13^C-NMR (75.47 MHz; CDCl_3_): δ 14.6 (4″-OCH_2_*C*H_3_); 63.4 (4″-O*C*H_2_CH_3_), 75.4 (C-2); 114.6 (C-3″,5″); 116.4 (C-8); 120.6 (C-α); 122.0 (C-4a); 122.1 (C-6); 123.6 (C-3′,5′); 125.0 (C-5); 126.2 (C-4); 126.4 (C-1″); 128.1 (C-2′,6′); 128.5 (C-2″,6″); 128.7 (C-3); 130.2 (C-7); 131.5 (C-β); 144.8 (C-1′); 147.8 (C-4′); 151.4 (C-8a); 159.4 (C-4″) ppm.

(*Z*)-4-Chloro-2-(4-nitrophenyl)-3-(4-nitrovinyl)-2*H*-chromene (**10i**). Orange solid, η 52%, m. p. 163–165 °C. ^1^H-NMR (300.13 MHz; CDCl_3_): δ 5.85 (s, 1H, H-2); 6.63 (d, 1H, H-α, *J* 12.2 Hz); 6.74 (d, 1H, H-β, *J* 12.2 Hz); 6.84 (dd, 1H, H-8, *J* 1.3 and 8.0 Hz); 7.01 (ddd, 1H, H-6, *J* 1.3, 7.3 and 8.0 Hz); 7.24 (ddd, 1H, H-7, *J* 1.3, 7.3 and 8.0 Hz); 7.36 (d, 2H, H-2′,6′, *J* 8.8 Hz); 7.56 (dd, 1H, H-5, *J* 1.3 and 8.0 Hz); 7.58 (d, 2H, H-2″,6″, *J* 8.8 Hz); 8.09 (d, 2H, H-3′,5′, *J* 8.8 Hz); 8.20 (d, 2H, H-3″,5″, *J* 8.8 Hz) ppm.^13^C-NMR (75.47 MHz; CDCl_3_): δ 77.0 (C-2); 116.6 (C-8); 120.8 (C-4a); 122.4 (C-6); 123.7 (C-3″,5″); 124.0 (C-3′,5′); 125.2 (C-5); 126.4 (C-3); 127.8 (C-α); 127.9 (C-2′,6′); 128.6 (C-4); 129.3 (C-2″,6″); 131.3 (C-7); 131.5 (C-β); 142.8 (C-1″); 145.3 (C-1′); 147.3 (C-4″); 147.9 (C-4′); 152.2 (C-8a) ppm. ESI^+^-MS *m/z* (%): 457.1 Cl^35^ [M *+* Na]^+^ (80); 459.1 Cl^37^ [M *+* Na]^+^ (15). EI-HRMS *m/z* calcd for (C_23_H_15_N_2_^35^ClO_5_) 434.0669, found 434.0668. Calcd for (C_23_H_15_N_2_^37^ClO_5_) 436.0640, found 436.0647.

(*E*)-4-Chloro-2-(4-nitrophenyl)-3-(4-nitrovinyl)-2*H*-chromene (**11i**). Orange solid, η 27%, m. p. 180–182 °C. ^1^H-NMR (300.13 MHz; CDCl_3_): δ 6.38 (s, 1H, H-2); 6.49 (d, 1H, H-β, *J* 16.5 Hz); 6.85 (dd, 1H, H-8, *J* 1.3 and 8.0 Hz); 7.01 (ddd, 1H, H-6, *J* 1.3, 7.6 and 8.0 Hz); 7.21 (ddd, 1H, H-7, *J* 1.3, 7.6 and 8.0 Hz); 7.57–7.62 (m, 5H, H-5, 2′,6′, 2″,6″); 7.75 (d, 1H, H-α, *J* 16.5 Hz); 8.16 (d, 2H, H-3′,5′, *J* 8.8 Hz); 8.20 (d, 2H, H-3″,5″, *J* 8.8 Hz) ppm. ^13^C-NMR: δ 75.4 (C-2); 116.9 (C-8); 121.6 (C-4a); 122.5 (C-6); 123.9 (C-3″,5″); 124.2 (C-3′,5′); 125.5 (C-4); 125.8 (C-5); 127.1 (C-α); 127.2 (C-2″,6″); 128.6 (C-2′,6′); 129.1 (C-β); 130.6 (C-3); 131.6 (C-7); 142.6 (C-1″); 144.3 (C-1′); 147.2 (C-4″); 148.1 (C-4′); 152.0 (C-8a) ppm.

### 3.6. General Procedure for the Synthesis of (Z- and E)-2-aryl-4-chloro-3-styrylquinoline-1(2H)-carbaldehydes ***12a**–**i/13a**–**i***

The appropriate phosphonium halide salt (**8a**–**c**, 0.20 mmol) in dry THF (20 mL) and sodium hydride (6 mg, 0.20 mmol) were placed in a previously dried flask. The mixture was heated at 60 °C for 15, 7 and 3 min to prepare the ylide **9a**, **9b** and **9c**, respectively. After these periods, the formylated derivative **2d**–**f** was added (0.07 mmol). The reaction mixture was refluxed or maintained at room temperature for the appropriate time depending of each formylated derivative (see Table 2). At the end, the reaction mixture was poured into water (40 mL) and ice (50 g), the pH adjusted to 3 with dilute hydrochloric acid. The reaction mixture was extracted with ethyl acetate (3 × 20 mL), the solvent was dried using anhydrous sodium sulphate and evaporated to dryness on the evaporator. The residue was purified by thin layer chromatography using mixtures of dichloromethane/hexane as eluent.

(*E*)-4-Chloro-2-phenyl-3-styrylquinoline-1(2*H*)-carbaldehyde (**12a**). Yellow solid, η 44% (11 mg), m.p. 72–74 °C. ^1^H-NMR (300.13 MHz; CDCl_3_): δ 6.80 (d, 1H, H-β, *J* 16.4 Hz); 6.97 (s, 1H, H-2); 7.02–7.05 (m, 1H, H-8); 7.20–7.35 (m, 10H, H-6,7, 3′,4′,5′,2″,3″,4″,5″,6″); 7.46 (m, 2H, H-2′,6′); 7.57 (d, 1H, H-α, *J* 16.4 Hz); 7.81–7.84 (m, 1H, H-5); 8.68 (s, 1H, 1-NC*H*O) ppm. ^13^C-NMR (75.47 MHz; CDCl_3_): δ 52.2 (C-2); 118.2 (C-8); 123.1 (C-β); 126.0 (C-5); 126.2 (C-6); 126.5 (C-4a); 127.0 (C-4); 127.1 (C-2′,6′); 127.5 (C-2″,6″); 128.3 (C-4′); 128.6 (C-4″); 128.8 (C-3′,5′,3″,5″); 129.6 (C-7); 131.4 (C-1′); 133.6 (C-α); 134.1 (C-3); 136.5 (C-1″); 137.2 (C-8a); 161.0 (1-N*C*HO) ppm. MS (ESI^+^) *m/z* (%): 394.1 ^35^Cl [M *+* Na]^+^ (100), 396.1 ^37^Cl [M *+* Na]^+^ (38). MS (ESI^+^) calcd for (C_24_H_18_^35^ClNO) 371.1077; found 371.1078. Calcd for (C_24_H_18_^37^ClNO) 373.1047; found 372.1048.

(*E*)-4-Chloro-3-(4-nitrostyryl)-2-phenylquinoline-1(2*H*)-carbaldehyde (**12c**). Orange solid, η 40% (12 mg), m.p. 243.5-245.3 °C. ^1^H-NMR (300.13 MHz; CDCl_3_): δ 6.81 (d, 1H, H-β, *J* 16.4 Hz); 6.96 (s, 1H, H-2); 7.04–7.07 (m, 1H, H-8); 7.23–7.32 (m, 7H, H-6,7, 2′,3′,4′,5′,6′); 7.59 (d, 2H, H-2′,6′, *J* 8.8 Hz); 7.71 (d, 1H, H-α, *J* 16.4 Hz); 7.85–7.88 (m, 1H, H-5); 8.19 (d, 2H, H-3″,5″, *J* 8.8 Hz); 8.67 (s, 1H, 1-NC*H*O) ppm. ^13^C-NMR (75.47 MHz; CDCl_3_): δ 52.1 (C-2); 118.3 (C-8); 124.1 (C-3″,5″); 125.9 (C-4a); 126.2 (C-6); 126.7 (C-5); 127.2 (C-β); 127.3 (C-4); 127.4 (C-2″,6″, 2′,6′); 128.6 (C-4′); 128.7 (C-3′,5′); 129.1 (C-3); 130.4 (C-7); 130.7 (C-α); 134.3 (C-8a); 136.8 (C-1′); 142.9 (C-1″); 147.2 (C-4″); 161.0 (1-N*C*HO) ppm. MS (ESI^+^) *m/z* (%): 417.9 ^35^Cl [M *+* H]^+^ (100), 419.9 ^37^Cl [M *+* H]^+^ (3). MS (ESI^+^) calcd for (C_24_H_17_^35^ClN_2_O_3_) 416.0928; found 416.0929. Calcd for (C_24_H_17_^35^ClN_2_O_3_) 418.0898; found 418.0100

(*E*)-4-Chloro-2-(4-methoxyphenyl)-3-styrylquinoline-1(2*H*)-carbaldehyde (**12d**). White solid, η 46% (13 mg), m.p. 173–175 °C. ^1^H-NMR (300.13 MHz; CDCl_3_): δ 3.72 (s, 3H, 4′-OC*H*_3_); 6.74 (d, 2H, H-3′,5′, *J* 8.8 Hz); 6.79 (d, 1H, H-β, *J* 16.4 Hz); 6.91 (s, 1H, H-2); 7.01–7.04 (m, 1H, H-8); 7.21 (d, 2H, H-2′,6′, *J* 8.8 Hz); 7.22–7.28 (m, 3H, H-3″,4″,5″); 7.33 (t, 2H, H-6,7, *J* 7.3 Hz); 7.45–7.48 (m, 2H, H-2″,6″); 7.54 (d, 1H, H-α, *J* 16.4 Hz); 7.80–7.84 (m, 1H, H-5); 8.67 (s, 1H, 1-NC*H*O) ppm. ^13^C-NMR (75.47 MHz; CDCl_3_): δ 51.9 (C-2); 55.2 (4′-O*C*H_3_); 114.1 (C-3′,5′); 118.2 (C-8); 123.0 (C-β); 125.9 (C-6); 126.2 (C-5); 126.5 (C-4a); 126.8 (C-4); 127.0 (C-3″,5″); 128.6 (C-4″); 128.7 (C-2′,6′); 128.8 (C-2″,6″); 129.5 (C-1′); 131.5 (C-3); 133.5 (C-α); 134.0 (C-8a); 136.6 (C-1″); 159.4 (C-4′); 160.9 (1-N*C*HO) ppm. MS (ESI^+^) *m/z* (%): 424.1 ^35^Cl [M *+* Na]^+^ (100); 426.1 ^37^Cl [M *+* Na]^+^ (37). MS (ESI^+^) calcd for (C_25_H_21_^35^ClNO_2_) 402.1255; found 402.1253. Calcd for (C_25_H_21_^37^ClNO_2_) 404.1255; found 402.1254.

(*E*)-4-Chloro-3-(4-ethoxystyryl)-2-(4-methoxyphenyl)quinoline-1(2*H*)-carbaldehyde (**12e**). Yellow solid, η 27% (8 mg), m.p. 234–236 °C. ^1^H-NMR (300.13 MHz; CDCl_3_):δ 1.41 (t, 3H, 4″-OCH_2_C*H*_3_, *J* 7,0 Hz); 3.70 (s, 3H, 4′-OC*H*_3_); 4.04 (q, 2H, 4″-OC*H*_2_CH_3_, *J* 7,0 Hz); 6.73 (d, 2H, H-3″,5″, *J* 8.8 Hz); 6.74 (d, 1H, H-β, *J* 16.2 Hz); 6.85 (d, 2H, H-3′,5′, *J* 8.8 Hz); 6.89 (s, 1H, H-2); 7.02–7.03 (m, 1H, H-8); 7.20 (d, 2H, H-2′,6′, *J* 8.8 Hz); 7.23–7.26 (m, 2H, H-6,7); 7.39 (d, 2H, H-2″,6″, *J* 8.8 Hz); 7.40 (d, 1H, H-α, *J* 16.2 Hz); 7.79–7.82 (m, 1H, H-5); 8.66 (s, 1H, 1-NC*H*O) ppm. ^13^C-NMR (75.47 MHz; CDCl_3_): δ 14.8 (4″-OCH_2_*C*H_3_); 51.8 (C-2); 55.1 (4′-O*C*H_3_); 63.5 (4″-O*C*H_2_CH_3_); 114.0 (C-3″,5″); 114.7 (C-3′,5′); 118.1 (C-8); 120.8 (C-α); 125.6 (C-1″); 125.9 (C-5); 126.0 (C-6); 126.6 (C-4a); 128.4 (C-2″,6″); 128.8 (C-2′,6′); 129.2 (C-7); 129.5 (C-1′) 131.9 (C-3); 133.2 (C-β,8a); 133.9 (C-4); 159.3 (C-4′); 159.5 (C-4″); 161.0 (1-N*C*HO) ppm. MS (ESI^+^) *m/z* (%): 445.9 ^35^Cl [M *+* H]^+^ (100); 447.9 ^37^Cl [M *+* H]^+^ (13).MS (ESI^+^) *m/z* calcd for (C_27_H_25_^35^ClNO_3_) 446.1516; found 446.1517. Calcd for (C_27_H_25_^37^ClNO_3_) 448.1516; found 446.1519.

(*E*)-4-Chloro-2-(4-methoxyphenyl)-3-(4-nitrostyryl)quinoline-1(2*H*)-carbaldehyde (**12f**). Yellow solid, η 53% (17 mg), m.p. 209–211 °C. ^1^H-NMR (300.13 MHz; CDCl_3_): δ 3.72 (4′-OC*H*_3_); 6.76 (d, 2H, H-3′,5′, *J* 8.7 Hz); 6.80 (d, 1H, H-β, *J* 16.1 Hz); 6.90 (s, 1H, H-2); 7.04–7.07 (m, 1H, H-8); 7.20 (d, 2H, H-2′,6′, *J* 8.7 Hz); 7.29–7.32 (m, 2H, H-6,7); 7.58 (d, 2H, H-2″,6″, *J* 8.7 Hz); 7.69 (d, 1H, H-α, *J* 16.1 Hz); 7.84–7.88 (m, 1H, H-5); 8.18 (d, 2H, H-3″,5″, *J* 8.7 Hz); 8.67 (s, 1H, 1-NC*H*O) ppm. ^13^C-NMR (75.47 MHz; CDCl_3_): δ 51.8 (C-2); 55.2 (C-4′); 114.2 (C-3′,5′); 118.3 (C-8); 124.1 (C-3″,5″); 126.0 (C-4a); 126.1 (C-6); 126.6 (C-5); 127.2 (C-β); 127.4 (C-2′,6′); 128.7 (C-2″,6″); 128.9 (C-1′); 129.6 (C-4); 130.4 (C-7); 130.6 (C-α); 130.8 (C-3); 134.3 (C-8a); 143.0 (C-1″); 147.2 (C-4″); 159.6 (C-4′); 160.9 (1-N*C*HO) ppm. MS (ESI*^+^*) *m/z* (%): 469.9 ^35^Cl [M *+* Na]^+^ (100); 471.9 ^37^Cl [M+Na]^+^ (2). MS (ESI^+^) calcd for (C_25_H_20_^35^ClN_2_O_4_) 447.1106; found 447.1110. Calcd for (C_25_H_20_^37^ClN_2_O_4_) 449.1106; found 448.1109.

(*E*)-4-Chloro-2-(4-nitrophenyl)-3-styrylquinoline-1(2*H*)-carbaldehyde (**12g**). Orange solid, η 30% (9 mg), m.p. 179.9–180.7 °C. ^1^H-NMR (300.13 MHz; CDCl_3_): δ 6.74 (d, 1H, H-β, *J* 16.4 Hz); 7.04 (s, 1H, H-2); 7.04–7.07 (m, 1H, H-8); 7.29–7.38 (m, 5H, H-3″,4″,5″,6,7); 7.46–7.49 (m, 4H, H-2′,6′, 2″,6″); 7.60 (d, 1H, H-α, *J* 16.4 Hz); 7.83–7.87 (m, 1H, H-5); 8.09 (d, 2H, H-3′,5′, *J* 8.8 Hz); 8.71 (s, 1H, 1-NC*H*O) ppm. ^13^C-NMR (75.47 MHz; CDCl_3_): δ 51.3 (C-2); 118.2 (C-8); 122.8 (C-β); 124.1 (C-3′,5′); 126.1 (C-4a); 126.5 (C-6); 126.54 (C-5); 127.1 (C-2′,6′); 127.7 (C-4); 128.6 (C-3′,5′); 128.9 (C-3″,5″); 129.0 (C-4″); 130.0 (C-7); 130.2 (C-8a); 133.5 (C-3); 133.7 (C-α); 136.1 (C-1′); 144.3 (C-1′); 147.8 (C-4′); 161.0 (1-N*C*HO) ppm. MS (ESI^+^) *m/z* (%): 417.1 ^35^Cl [M *+* H]^+^ (30); 419.1 ^37^Cl [M *+* H]^+^ (2). MS (ESI^+^) calcd for (C_24_H_18_^35^ClN_2_O_3_) 417.0996; found 417.0999. Calcd for (C_24_H_18_^37^ClN_2_O_3_) 419.0996; found 417.0997.

(*E*)-4-Chloro-3-(4-ethoxystyryl)-2-(4-nitrophenyl)quinoline-1(2*H*)-carbaldehyde (**12h**). Yellow oil, η 24%. ^1^H-NMR (300.13 MHz; CDCl_3_): δ 1.42 (t, 3H, 4″-OCH_2_C*H*_3_, *J* 7.0 Hz); 4.04 (q, 2H, 4″-OC*H*_2_CH_3_, *J* 7.0); 6.69 (d, 1H, H-β, *J* 16.4 Hz); 6.86 (d, 2H, H-3″,5″, *J* 8.7 Hz); 7.01 (s, 1H, H-2); 7.03–7.06 (m, 1H, H-8); 7.27–7.31 (m, 2H, H-6,7); 7.40 (d, 2H, H-2″,6″, *J* 8.7 Hz); 7.47 (d, 2H, H-2′,6′, *J* 8.7 Hz); 7.47 (d, 1H, H-α, *J* 16.4 Hz); 7.81–7.84 (m, 1H, H-5); 8.08 (d, 2H, H-3′,5′, *J* 8.7 Hz); 8.70 (s, 1H, 1-NC*H*O) ppm. ^13^C-NMR (75.47 MHz; CDCl_3_): δ 14.8 (4″-OCH_2_*C*H_3_); 51.3 (C-2); 63.6 (4″-O*C*H_2_CH_3_); 114.8 (C-3″,5″); 118.2 (C-8); 120.5 (C-α); 124.1 (C-3′,5′); 126.2 (C-4a); 126.3 (C-5); 126.4 (C-6); 128.5 (C-2″,6″); 128.6 (C-2′,6′); 128.7 (C-1″); 129.7 (C-7); 130.5 (C-3); 133.3 (C-β); 133.5 (C-4); 144.4 (C-1′); 147.7 (C-4′); 159.8 (C-4″); 161.1 (1-N*C*HO) ppm. MS (ESI^+^) *m/z* (%): 461.1 ^35^Cl [M *+* H]^+^ (50); 463.1 ^37^Cl [M *+* H]^+^ (3).MS (ESI^+^) *m/z* calcd for (C_26_H_22_^35^ClN_2_O_4_) 461.1263; found 461.1261. Calcd for (C_26_H_22_^37^ClN_2_O_4_) 463.1263; found 463.1263.

### 3.7. Theoretical Studies

Theoretic studies were performed get an insight into the selectivity of the Wittig reaction with quinoline-3-carbaldehydes **2d**–**f**. All relevant structures were optimized using density functional theory (DFT). Gaussian 09 et al. [33] was used for all single-point calculations employing the standard fine grid and a B3LYP/6-31G(d) [34,35,36] level of theory. This is in agreement with the literature that suggests that larger basis sets do not cause significant changes in the relative energies of the two diastereomeric transition states, here in study. The use of THF as solvent was simulated using a standard PCM model. Vibrational frequency calculations were performed to ensure the optimized structures represented the local minima. All transition states here shown contained a single negative frequency, and the corresponding eigenvector was verified to correspond to the expected reaction coordinate. IRC calculations were also performed and lead to the formation of the next intermediate.

## 4. Conclusions

This work establishes that 2-aryl-4-chloro-2*H*-chromene-3-carbaldehydes are useful building blocks and afforded several new derivatives in good yields. Interesting heterocycles, such as 3*H*-chromeno[3,4-*c*]quinolines, were obtained, and it also demonstrated that they could be used in Wittig reactions to get new (*Z* and *E*)-2-aryl-4-chloro-3-styryl-2*H*-chromenes. It was evident that 2-aryl-4-chloro-1,2-dihydroquinoline-1,3-dicarbaldehydes are less reactive. However, it was also possible to use them in Wittig reactions, where they showed high selectivity towards the (*E*)-2-aryl-4-chloro-3-styrylquinoline-1(2*H*)-carbaldehydes. This unique selectivity was explained through density functional theory analysis.

## Figures and Tables

**Table 1:** Optimization of reaction conditions for the synthesis of (*Z* and *E*)-2-aryl-4-chloro-3-styryl-2*H*-chromenes **10a**–**f** and **11a**–**i**.

Entry	Comp. 2	Ylide	NaH ^a^ (Equiv)	1st Step ^b^ (Time)	2nd Step ^c^ (Time)	(*Z*)-isomer ^d^ (Yield, %)	(*E*)-isomer ^d^ (Yield, %)
1	**2a** (R=H)	**9a**	5	3 h	3 h	**10a**, 18	**11a**, 10
2			7	3 h	3 h 30 min	**10a**, 25	**11a**, 19
3			7	1.5 h	21 h	**10a**, 32	**11a**, 5
4			7	20 min	24 h	**10a**, 32	**11a**, 25
5		**9b**	7	10 min	3 h 45 min	**10b**, 45	**11b**, 25
6		**9c**	7	3 min	24 h	**10c**, 41	**11c**, 33
7	**2b** (R=OCH_3_)	**9a**	7	15 min	3 h 50 min	**10d**, 50	**11d**, 28
8		**9b**	8	5 min	3 h 30 min	**10e**, 50	**11e**, 20
9		**9c**	8	3 min	3 h	**10f**, 53	**11f**, 35
10	**2c** (R=NO_2_)	**9a**	8	15 min	5 h	**10g**, -	**11g**, -
11			8	15 min	3 h	**10g**, -	**11g**, -
12			9	10 min	24 h ^f^	**10g**, 27	**11g**, -
13			3	10 min	40 min ^f^	**10g**, 34	**11g**, 15
14		**9b**	7	2 min	4 h	**10h**, 20	**11h**, -
15			7	3 min	1 h ^f^	**10h**, 45	**11h**, 24
16		**9c**	8	6 min	4 h	**10i**, -	**11i**, -
17			3	10 min ^e^	2 h^f^	**10i**, 52	**11i**, 27

^a^ Molar excess; ^b^ Time to obtain the ylide; ^c^ Time after the aldehyde addition; ^d^ Isolated compound yield in %; ^e^ THF freshly dried; ^f^ Second step at room temperature.

**Table 2:** Optimization of reaction conditions for the synthesis of (*E*)-2-aryl-4-chloro-3-styrylquinoline-1(2*H*)-carbaldehydes **12a**–**i**.

Entry	Comp. 2	Ylide	2nd step ^a^ (Time)	Comp. 12	(*E*)-Isomer ^b^ (Yield, %)
1	**2d** (R=H)	**9a**	50 min	**12a**	35
2			1 h 20 min	**12a**	40
3			2 h	**12a**	44
4		**9b**	46 min	**12b**	17 ^c^
5			1 h 30 min	**12b**	35 ^c^
6		**9c**	2 h	**12c**	33
7			3 h	**12c**	40
8	**2e** (R=OCH_3_)	**9a**	45 min	**12d**	46
9			1 h 20 min	**12d**	35
10			2 h 20 min	**12d**	20
11		**9b**	40 min	**12e**	27
12			1 h 20 min	**12e**	25
13		**9c**	2 h	**12f**	43
14			3 h	**12f**	53
15	**2f** (R=NO_2_)	**9a**	58 min	**12g**	18
16			2 h 20 min	**12g**	30
17		**9b**	45 min	**12h**	24
18		**9c**	55 min	**12i**	-
19			2 h	**12i**	27^c^

^a^ Time after the aldehyde addition; ^b^ Isolated compound yield in %; ^c^ Mixture of the two isomers.
